# Elevated α-1,2-mannosidase MAN1C1 in glioma stem cells and its implications for immunological changes and prognosis in glioma patients

**DOI:** 10.1038/s41598-024-72901-2

**Published:** 2024-09-27

**Authors:** Don Carlo Batara, Hyun-Jin Kim, Le Thi Phan, Minseo Kim, Young-Ok Son, Seongsoo Lee, Sang-Ik Park, Young Sun Choi, Samuel Beck, Sung-Hak Kim

**Affiliations:** 1 Animal Molecular Biochemistry Laboratory, Department of Animal Science, College of Agriculture and Life Sciences, Chonnam National University, Gwangju, 61186 Republic of Korea; 2 Computational Biology and Bioinformatics Laboratory, Department of Integrative Biotechnology, Sungkyunkwan University, Suwon, Gyeonggi-do 16419 Republic of Korea; 3 Department of Animal Biotechnology, Faculty of Biotechnology, College of Applied Life Sciences, Interdisciplinary Graduate Program in Advanced Convergence Technology and Science, Jeju National University, Jeju, 63243 Republic of Korea; 4 Gwangju Center, Korea Basic Science Institute (KBSI), 49, Dosicheomdansaneop-ro, Nam‐gu, Gwangju, 61751 Republic of Korea; 5 Department of Systems Biotechnology, Chung-Ang University, Anseong-si, Gyeonggi-do 17546 Republic of Korea; 6 Laboratory of Veterinary Pathology, College of Veterinary Medicine and BK21 Plus Project Team, Chonnam National University, Gwangju, 61186 Republic of Korea; 7 Jeollanam-do Agriculture Research and Extension Services Livestock Research Institute, Naju-si, Jeollanam-do 58213 Republic of Korea; 8 Department of Dermatology, Center for Aging Research, Chobanian & Avedisian School of Medicine, Boston University, Boston, 02118 USA

**Keywords:** MAN1C1, Glioblastoma, Glioma stem cell, Immune, Biomarker, Transcriptomics, Biochemistry, Cancer, Cell biology, Immunology, Molecular biology, Neuroscience, Stem cells, Biomarkers, Medical research, Molecular medicine, Neurology, Oncology

## Abstract

Glioblastoma multiforme (GBM) is the most aggressive type of primary brain tumor, and the presence of glioma stem cells (GSCs) has been linked to its resistance to treatments and recurrence. Additionally, aberrant glycosylation has been implicated in the aggressiveness of cancers. However, the influence and underlying mechanism of N-glycosylation on the GSC phenotype and GBM malignancy remain elusive. Here, we performed an in-silico analysis approach on publicly available datasets to examine the function of N-glycosylation-related genes in GSCs and gliomas, accompanied by a qRT-PCR validation experiment. We found that high α-1,2-mannosidase MAN1C1 is associated with immunological functions and worse survival of glioma patients. Differential gene expression analysis and qRT-PCR validation revealed that MAN1C1 is highly expressed in GSCs. Furthermore, higher MAN1C1 expression predicts worse outcomes in glioma patients. Also, MAN1C1 expression is increased in the perinecrotic region of GBM and is associated with immunological and inflammatory functions, a hallmark of the GBM mesenchymal subtype. Further analysis confirmed that MAN1C1 expression is closely associated with infiltrating immune cells and disrupted immune response in the GBM microenvironment. These suggest that MAN1C1 is a potential biomarker for gliomas and may be important as an immunotherapeutic target for GBM.

**Supplementary Information:**

The online version contains supplementary material available at 10.1038/s41598-024-72901-2.

## Introduction

Glioblastoma multiforme (GBM) is the most aggressive form of gliomas, with an incidence of 3–6 per 100,000 persons each year and a 14.6-month post-diagnosis survival time despite several treatment options^1–3^. The malignant phenotype of GBM is associated with the presence of GSCs. GSCs can grow in complex and harsh microenvironmental niches, self-renew, duplicate the heterogeneity of the parent tumor, and promote treatment resistance^4–6^. In the past years, aberrant protein glycosylation has been also noted to play a role in the progression and metastasis of various cancers including GBM and cancer stem cells (CSC)^7–13^. Notably, the majority of cell surface proteins are heavily glycosylated, which is necessary for them to function as intended^14^. However, aberrant modification to the glycan structures of these surface proteins in many cancers contributes to increased proliferation, invasion, migration, stem cell retention, EMT activation, and immune evasion^11,15^.

N-glycosylation is an intricate process that occurs during translation, where glycan structures are attached to the amino group of asparagine (Asn) residues^16,17^. This process begins in the ER, where the flippase flips the dolichol phosphate-bound oligosaccharide (GlcNAc2Man5) precursor from the cytosol to the ER luminal side. Next, four mannose and three glucose molecules are added to the GlcNAc2Man5-dolichol phosphate to create Glc3Man9GlcNAc2-PP-dolichol. The oligosaccharyltransferase (OST) complex subsequently transfers this oligosaccharide to the N-X-S-T sequence of a nascent peptide chain. Following the removal of three terminal glucose and one mannose, the newly formed glycopeptide exits the ER and enters the Golgi apparatus^18^. Next, the α-1,2-mannosidases (MAN1A1, MAN1A2, MAN1B1, and MAN1C1) trim the mannose residues of nascent glycoproteins from high-mannose glycans (Man8–9GlcNAc2) into 5-mannose glycans (Man5GlcNAc2). The trimming of mannose residues provides a substrate for several glycosyl transferases to facilitate the synthesis of high-mannose, hybrid, or complex branched tri- or tetra-antennary N-glycans (Supplementary Fig. S1A)^19,20^. Matrisome and glycoproteomic analysis indicated that high-grade glioma cells consist of highly complex multi-antennary fucosylated and sialylated N-glycans, which are associated with their malignant characteristics^21^. Further, spatially resolved glycoproteomics profiles in canine gliomas reveal that a high-mannose N-linked glycan was enriched in benign regions, and biantennary complex N-linked glycan was enhanced in necrotic regions^22^.

Various studies have investigated the role of α1,2-mannosidases expression in different types of cancer. For instance, low expression of MAN1A1 preserves the slow-cycling state and tumorigenicity of CD133 + GSCs^23^, and reduces adhesion capabilities breast cancer cells, leading to a poorer prognosis^17^, whereas high expression of MAN1A1 has been associated with a poor prognosis in hepatocarcinoma^25^ and ovarian cancer^16^. On the other hand, elevated expression of MAN1C1 has been noted in acute lymphoblastic leukemia and non-Hodgkin lymphoma^26,27^, while downregulated in clear cell renal cell carcinoma, hepatocarcinoma, and intrahepatic cholangiocarcinoma^24,25,28^. Recently, several N-glycosylation genes including MAN1A1 and MAN1C1, were found to be highly expressed GBM mesenchymal subtype^11^. Despite the knowledge of the regulatory effect of 1,2-mannosidases on tumor progression, its potential molecular mechanism in GBM, especially in GSCs, is still unclear.

In this study, we performed an integrated transcriptomic analysis using clinical, bulk, spatial, and single-cell RNA sequencing data to explore the role of N-glycosylation genes in gliomas. We demonstrated that increased MAN1C1 expression in GSCs correlates with immunological changes and predicts poor outcomes in patients with gliomas. Our findings provide new insights into the molecular role of MAN1C1 and suggest that MAN1C1 could be a therapeutic target in gliomas.

## Results

### MAN1C1 is differentially expressed in GSCs

**Fig. 1:**
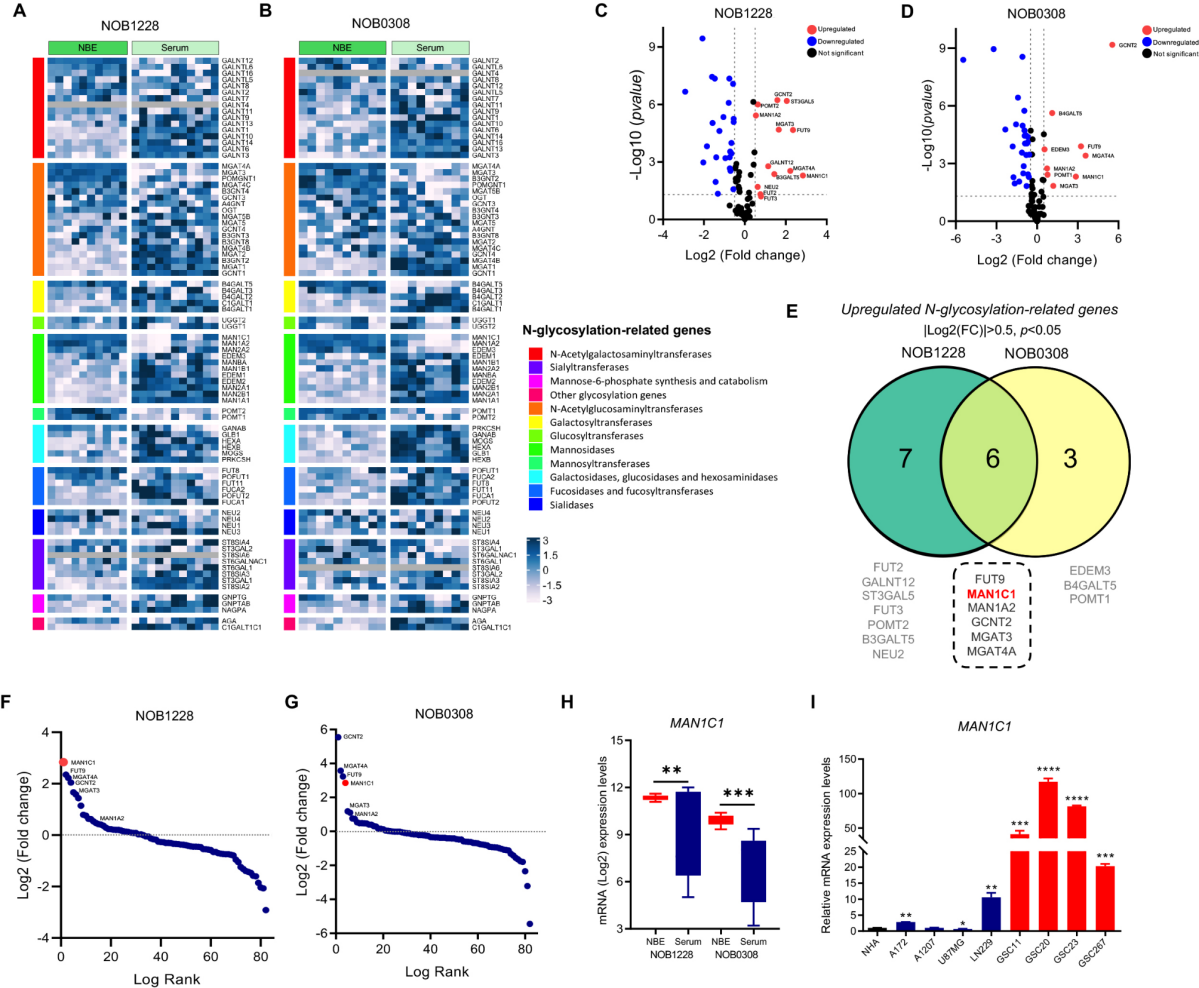
MAN1C1 is differentially expressed in GSCs. (**A**,**B**) Complex heatmap illustrating the differential expression of 83 N-glycosylation related genes in (**A**) NOB1228 and (**B**) NOB0308 GSCs under NBE and FBS culture condition in GSE4536 dataset; (**C**,**D) **Volcano plots depicting the differentially expressed N-glycosylation related genes in (**C**) NOB1228 and (**D**) NOB0308 GSCs under NBE and FBS culture condition in GSE4536 dataset; (**E) **The top N-glycosylation related genes (Log2(FC) > 0.5, *p *value < 0.05) from the NOB1228 and NOB0308 are cell lines displayed in a Ven diagram. (**F**,**G**) Log-rank fold-change for upregulated N-glycosylation-related genes in (**F**) NOB1228 and (**G**) NOB0308 GSCs. (**H) **mRNA expression levels of MAN1C1 in the GSE4536 dataset; (**I) **RT-qPCR analysis of the MAN1C1 basal mRNA levels in GBM cell lines (A172, A1207, U87MG, LN229), GSC lines (GSC11, GSC20, GSC23, GSC267), and NHA cells.

The GSE4536 dataset was used to assess the differentially expressed N-glycosylation-related genes in GSCs under stem cell (NBE) and differentiated (serum) culture conditions (Fig. 1A,B). Among the 83 N-glycosylation genes, 13 and 9 genes were significantly elevated in NOB1228 and NOB0308, respectively, under NBE conditions (Fig. 1C,D). After filtering, six of the genes (FUT9, MAN1C1, MAN1A2, GCNT2, MGAT3, and MGAT4A) were found to be common in the NBE cultures (Fig. 1E). We opted to choose MAN1C1 as a potential gene because it is essential for trimming mannose residues from precursor oligosaccharides and for the transformation of high-mannose N-glycans into hybrid and complex-type structures^20^. Notably, MAN1C1 was a highly expressed N-glycosylation-related gene based on its log-rank value (Fig. 1F,G). The MAN1C1 mRNA expression level in stem cells was significantly greater than that in the differentiated cells (Fig. 1H). We confirmed this result through qRT-PCR analysis of MAN1C1 basal mRNA expression levels in normal astrocytes, GBM, and GSCs lines. GSCs showed much greater MAN1C1 expression than GBM and normal astrocytes (Fig. 1I). Similarly, expression levels of other mannosidases (MAN1A1, MAN1A2, and MAN1B1) are highly upregulated in mesenchymal GSCs (GSC20, GSC267) compared to GBM cells and normal astrocytes (Supplementary Fig. S1B–D). These findings indicate that MAN1C1 is differentially expressed in GSCs.

### MAN1C1 is highly expressed in GBM and is correlated with poor outcomes in glioma patient

**Fig. 2:**
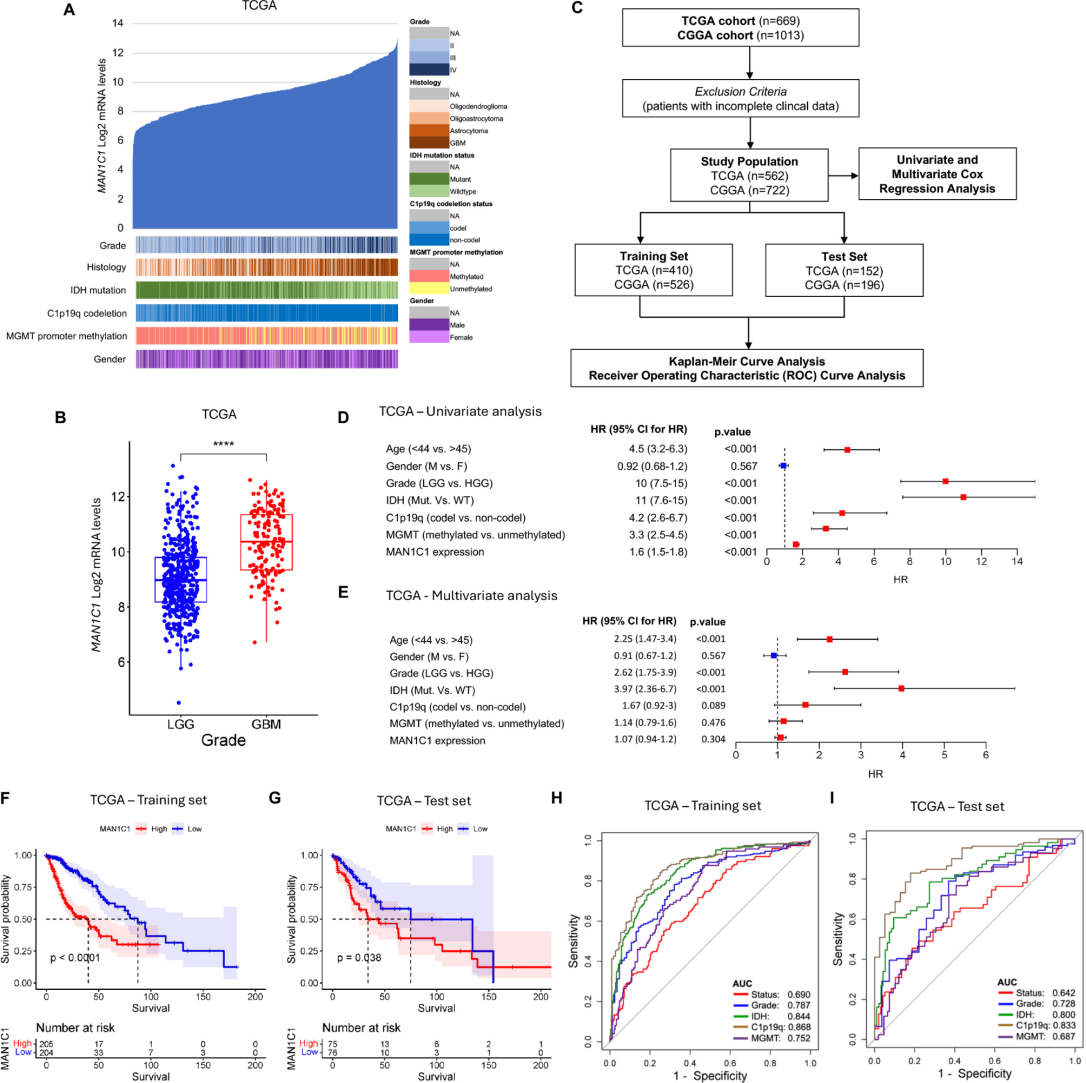
MAN1C1 is highly expressed in GBM and correlated to poor prognosis of glioma patients. (**A**) MAN1C1 mRNA expression and clinical features in the TCGA dataset (*n* = 669); (**B**) Raincloud plot of MAN1C1 expression in GBM and LGG patients. (**C**) Flow chart showing the patient population in the TCGA and CGGA cohorts; (**D**,**E**) Univariate and multivariate Cox proportional hazards regression analysis of variables influencing overall patient survival in the TCGA dataset. (**F**,**G**) Kaplan–Meier analysis of glioma patients with MAN1C1 High/Low expression in (**F**) training and (**G**) test set in the TCGA dataset. (**H**,**I**) ROC curve and the risk score distribution stratified by MAN1C1 High/Low expression in (**H**) training and (**I**) test set in the TCGA dataset.

We then compared MAN1C1 expression patterns between TCGA and CGGA datasets. We demonstrated that MAN1C1 was significantly expressed in high-grade gliomas (Fig. 2A,B, Supplementary Fig. S2A,B). Furthermore, MAN1C1 expression is elevated in glioma patients with wild-type IDH, 1p/19q co-deletion, and an unmethylated MGMT promoter status. Patients with incomplete or missing clinical data were excluded before subsequent analysis (Fig. 2C). Univariate and multivariate analyses revealed that MAN1C1^High^ expression was an independent predictor of overall survival (OS) in glioma patients (Fig. 2D,E, Supplementary Fig. S2C,D). Other independent prognostic variables included older age (> 45 years), high-grade glioma, wild-type IDH, 1p/19q co-deletion, and unmethylated MGMT promoter status. To avoid bias, we randomly separated the datasets into training and test sets before performing the KM survival and ROC analyses. KM curve analysis revealed that patients with elevated MAN1C1 expression had a worse OS rate (Fig. 2F,G, Supplementary Fig. S2E,F). The area under the ROC curve (AUC) in the training (0.690) and test (0.642) cohorts had satisfactory predictive values in the TCGA dataset (Fig. 2H–I). This finding was confirmed in the CGGA cohort (Supplementary Fig. S2G,H). Additionally, we used GBM samples from the TCGA, CGGA, and Gravendeel datasets for survival and univariate analyses. The results confirmed that higher MAN1C1 expression was associated with shorter OS in GBM patients (Supplementary Fig. S3A–D). Hence, these results suggest that elevated MAN1C1 expression may be a predictor of prognosis and OS in glioma patients.

### MAN1C1 is a highly expressed GBM-MES subtype and is enriched in the GBM perinecrotic region

**Fig. 3:**
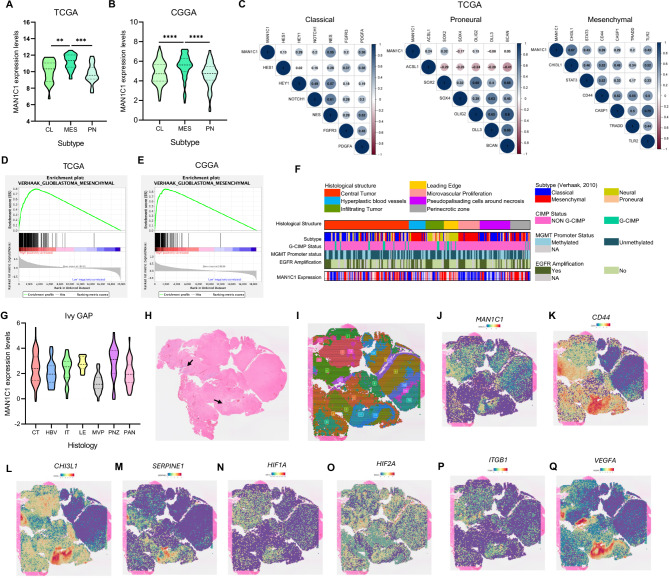
MAN1C1 is highly expressed in the mesenchymal GBM and enriched in the perinecrotic GBM region. (**A**,**B**) MAN1C1 mRNA levels in GBM subtypes from the (**A**) TCGA and (**B**) CGGA dataset (*CL *Classical, *ME *Mesenchymal, *PN *Proneural); (**C**) Correlation of MAN1C1 expression with CL, MES, and PN markers in the TCGA dataset. (**D**,**E**) GSEA of VERHAAK_GLIOBLASTOMA_MESENCHYMAL in (**D**) TCGA and (**E**) GCCA dataset; (**F**) Heatmap of MAN1C1 expression and clinical data in the Ivy GAP dataset; (**G**) MAN1C1 mRNA levels in various GBM regions as described in the IvyGAP dataset. (*LE* leading edge, *IT *infiltration tumor; *CT *cellular tumor, *PNZ *perinecrotic zone, *PAN *pseudopalisading cells around necrosis, *HBV *hyperplastic blood vessels, *MVP *microvascular proliferation); (**H**) Coronal tissue section H&E histology staining (arrow indicates perivascular and necrotic region); (**I**) Spatial clusters based on gene expression in the coronal tissue section; (**J**–**P**) SpatialFeaturePlot of (**J**) MAN1C1, mesenchymal genes (**K**) CD44, (**L**) CHI3L1, (**M**) SERPINE1, hypoxia genes (**N**) HIF1A and (**N**) HIF2A, and perivascular genes (**O**) ITGB1 and (**P**) VEGFA.

We used TCGA and CGGA data to identify GBM subtypes that express MAN1C1. We demonstrated that the GBM-MES subtype expressed MAN1C1 at high levels (Fig. 3A,B). MAN1C1 was linked to MES markers according to correlation analysis and GSEA (Fig. 3C–E). We further confirmed the expression levels of MAN1C1 in GSC11 and GSC20, revealing that MAN1C1 is strongly expressed in GSC20 cells (Supplementary Fig. S4A). GSC11 cells have a proneural gene signature (CD133+), whereas GSC20 shows a mesenchymal gene signature (CD44+) (Supplementary Fig. S4B,C)^29^. Next, we identified the anatomical region of the GBM that promotes MAN1C1 expression using the Ivy-GAP dataset. The result indicates that the perinecrotic GBM region had elevated MAN1C1 expression (Fig. 3F,G). To confirm these results, we conducted a spatial transcriptomic analysis. The tumor area with vascular growth spreading from the necrotic area was evident in the H&E-stained image (Fig. 3H). Clustering analysis revealed that the spatial segments could be classified into 21 clusters (Fig. 3I). MAN1C1 was significantly expressed in clusters 11 and 20 (Fig. 3J), which contained vascular and necrotic regions, respectively. Along with the perivascular GBM niche, the necrotic zones of GBM may serve as neurogenic niches for the formation of CSCs^30^, where hypoxia plays a fundamental role^31^. This region is suitable for GBM-MES subtypes. We also demonstrated that MAN1C1 was co-expressed with perivascular, hypoxic, and MES markers (Fig. 3K–Q). Additionally, functional analysis of MAN1C1-enriched clusters revealed associations with signal transduction, apoptosis, inflammatory and immunological responses, and cell adhesion and migration (Supplementary Fig. S4D,E). Collectively, these findings suggest that MAN1C1 may influence the aggressive phenotype of MES-subtype in patients with GBM.

### MAN1C1 is involved in immune responses and controls tumor-related signaling pathways in gliomas

**Fig. 4:**
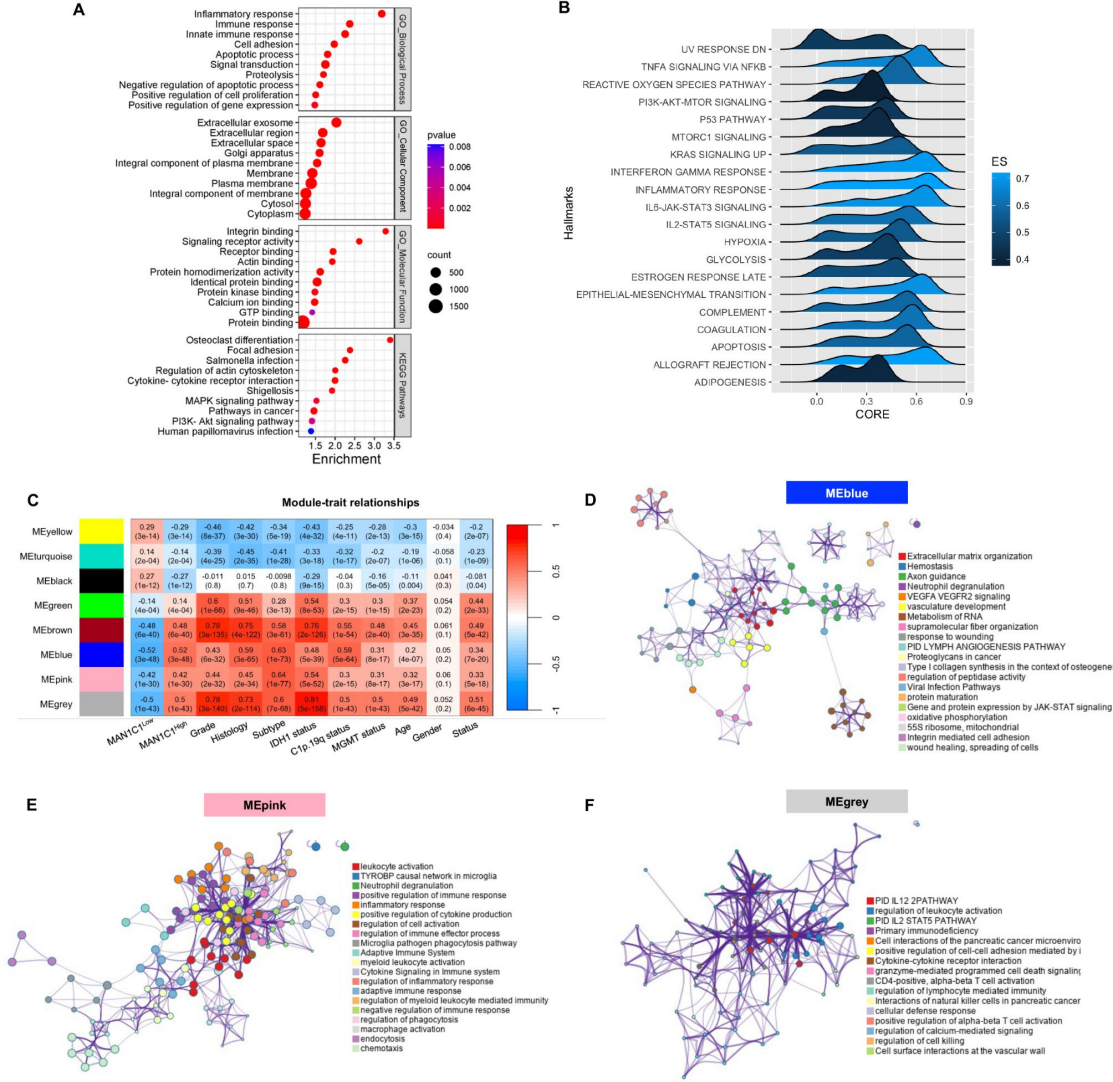
MAN1C1 expression is associated with immune-related genes in gliomas. (**A**) Dot plot of the functional enrichment result of MAN1C1 correlated genes in the TCGA dataset; (**B**) Ridge plot of the GSEA cancer hallmarks correlated with high MAN1C1 in the TCGA dataset; (**C**) Correlation between MAN1C1 expression and clinical information analyzed by WGCNA. (**D–F**) Enrichment ontology of gene hubs in the (**D**) MEBlue, (**E**) MEpink, and (**F**) MEgrey.

Using the TCGA dataset, we performed a correlation analysis to evaluate the associated molecular functions and signaling pathways associated with MAN1C1 expression. Functional enrichment analysis of the correlated genes revealed that MAN1C1 expression is associated with the innate immune response, cell migration, adhesion, cell proliferation, and signal transmission (Fig. 4A). MAN1C1 expression is also linked to cellular components, such as the plasma membrane and Golgi apparatus, as well as molecular functions, including integrin binding and signal receptor activation. Furthermore, MAN1C1 expression was correlated with KEGG pathway genes involved in focal adhesion and cytokine-cytokine receptor interactions. Next, we classified the dataset as MAN1C1^High/Low^ based on the median mRNA expression levels and performed GSEA. We found that MAN1C1 was related to immunological and inflammatory responses, hypoxia, EMT, and cancer signaling pathways (p53, TGF-beta, IL6/JAK-STAT3, IL2/STAT5) (Fig. 4B). Similar results were obtained for the TCGA_GBM cohorts (Supplementary Fig. S5A,B).

To further understand the role of MAN1C1 expression in gliomas, we conducted WGCNA on the TCGA dataset. After removing outliers, the analysis included 668 samples (Supplementary Fig. S5C). A soft threshold (β) = 10 was used to assess adjacency (Supplementary Fig. S5D). Genes related to MAN1C1^High/Low^ were identified and organized into eight gene modules (Supplementary Fig. S5E). The MEbrown, MEblue, MEpink, and MEgray gene modules were strongly correlated (*R* > 0.4; *p* ≤ 0.05) with MAN1C1^High^ expression (Fig. 4C). Functional annotation suggested that the gene hubs of MEbrown (229 genes) are linked to the cell cycle. Moreover, gene hubs in MEblue (421 genes), MEpink (489 genes), and MEgray (33 genes) were associated with cell adhesion, immunological and inflammatory responses, and cell cell interactions (Fig. 4D,E). Overall, these findings suggest that MAN1C1 may play a role in immune responses and regulate tumor-related signaling pathways in gliomas.

### Associations between MAN1C1 and immune cells

**Fig. 5:**
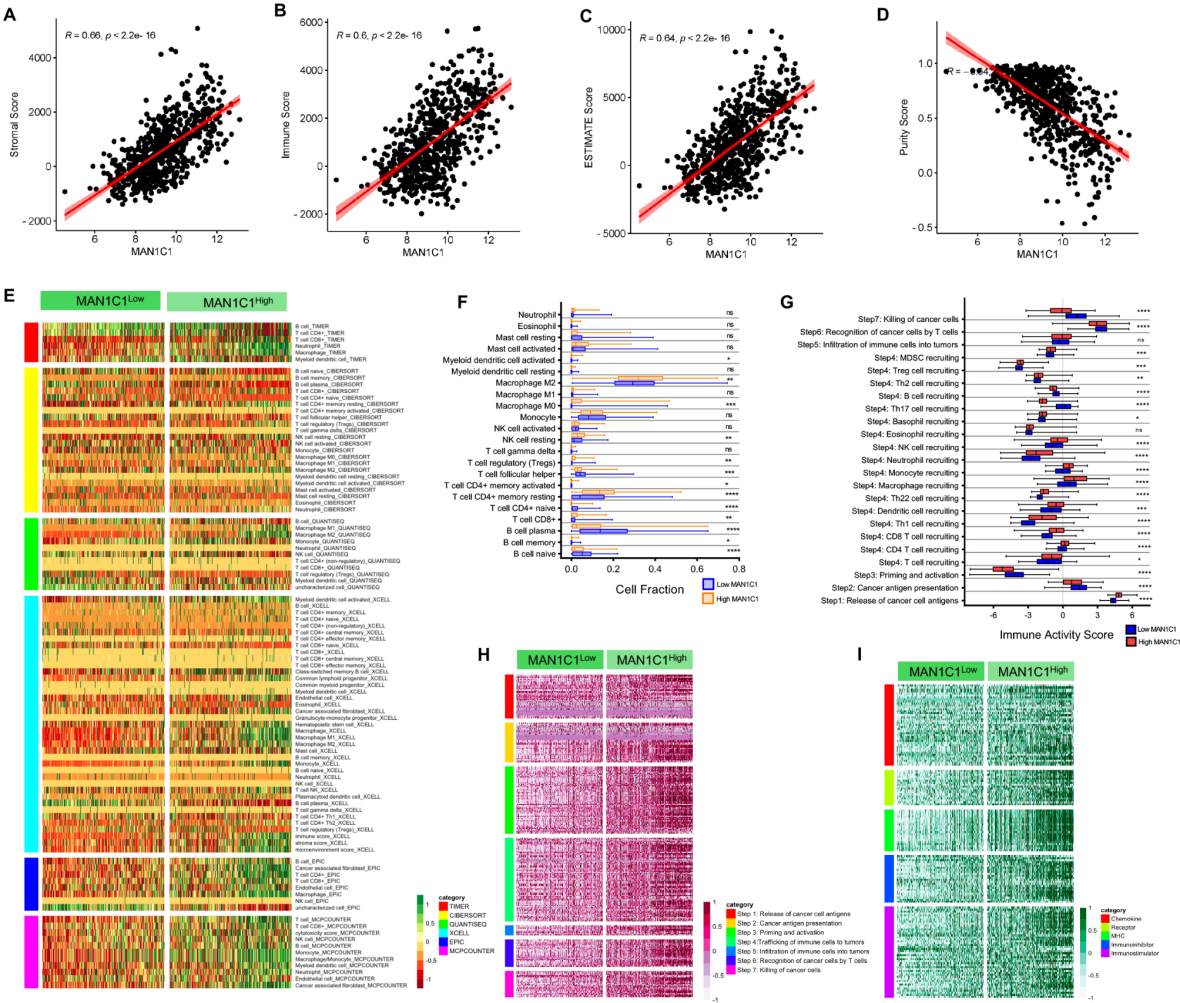
MAN1C1 expression correlates with increased immune cell infiltration in gliomas. (**A–D**) Correlations between (**A**) stromal score, (**B**) immune score, (**C**) ESTIMATE score, and (**D**) tumor purity and MAN1C1 expression levels; (**E**) A heatmap of immune responses according to TIMER, CIBERSORT, QUANTISEQ, XCELL, MCP counter, and EPIC algorithms between the high- and low-MAN1C1 groups of gliomas in the TCGA dataset; (**F**) Differences in immune cells and function in CIBERSORT between high- and low-MAN1C1 groups of gliomas in the TCGA dataset. (**G**) Differences in the Immune activity scores cancer-immunity cycle between high- and low-MAN1C1 groups of gliomas in the TCGA dataset. (**H**) A heatmap of gene signatures in the cancer-immunity cycle between the high- and low-MAN1C1 groups of gliomas in the TCGA dataset; (**I**) A heatmap of immunomodulator and chemokine-related genes between the high- and low-MAN1C1 groups of gliomas in the TCGA dataset;.

To better understand the relationship between MAN1C1 expression and the tumor microenvironment (TME), we computed correlations between MAN1C1 expression and stromal, immune, ESTIMATE, and tumor purity scores. MAN1C1 expression was positively correlated (*R* > 0.6; *p* ≤ 0.01) with the stromal, immune, and ESTIMATE scores (Fig. 5A–C). In contrast, tumor purity was inversely associated with MAN1C1 expression (Fig. 5D). These findings suggest that the proportion of infiltrating immune cells increases in parallel with elevated MAN1C1 expression in gliomas. Next, we used TIMER 2.0 to identify the different types of tumor-infiltrating cells (TICs). Our findings demonstrated a strong correlation between MAN1C1 expression and TICs, including myeloid dendritic cells, CD4 + T cells, neutrophils, MO/M2 macrophages, monocytes, and CD4 + T cells (Fig. 5E). Using the CIBERSORT cohort, we showed that gliomas with MAN1C1^High^ expression exhibited increased infiltration of immune cells, including neutrophils, MO/M2 macrophages, and CD4 + T cells, and decreased infiltration of CD8 + T cells (Fig. 5F).

The functions of the chemokine system and other immunomodulators are directly influenced by the activity of the cancer immune system^32^. We then examined the association between MAN1C1 expression and the seven-step cancer-immunity cycle in the TCGA cohort (Fig. 5G). The MAN1C1^High^ samples exhibited an increase in the release of cancer cell antigens (Step 1) and the trafficking of immune cells, including T cells, CD4 + T cells, CD8 + T cells, Th1 cells, DCs, Th22 cells, MO, monocytes, neutrophils, NK cells, basophils, Tregs, and MDSCs, to tumors (Step 4). There was no significant difference in immune cell infiltration (Step 5) between MAN1C1^High^ and MAN1C1^Low^ samples. Interestingly, MAN1C1^Low^ samples performed much better in terms of cancer antigen presentation (Step 2), priming and activation (Step 3), T-cell recognition (Step 6), and cancer cell killing (Step 7).

Finally, we examined the association between MAN1C1 expression and the expression of genes at different stages of the cancer-immunity cycle from the TIP website and between MAN1C1 expression and the expression of immunomodulator and chemokine genes from the TISIDB website. The results revealed that when MAN1C1 expression increased, the genes in various stages of the cancer-immune cycle also increased (Fig. 5H). Similarly, the upregulation of MAN1C1 activated immunoinhibitors, immunomodulators, MHC molecules, receptors, and ligands (Fig. 5I). Overall, our findings indicate that elevated MAN1C1 expression may contribute to immune cell infiltration in gliomas.

### Construction of the MAN1C1-IPS risk model

**Fig. 6:**
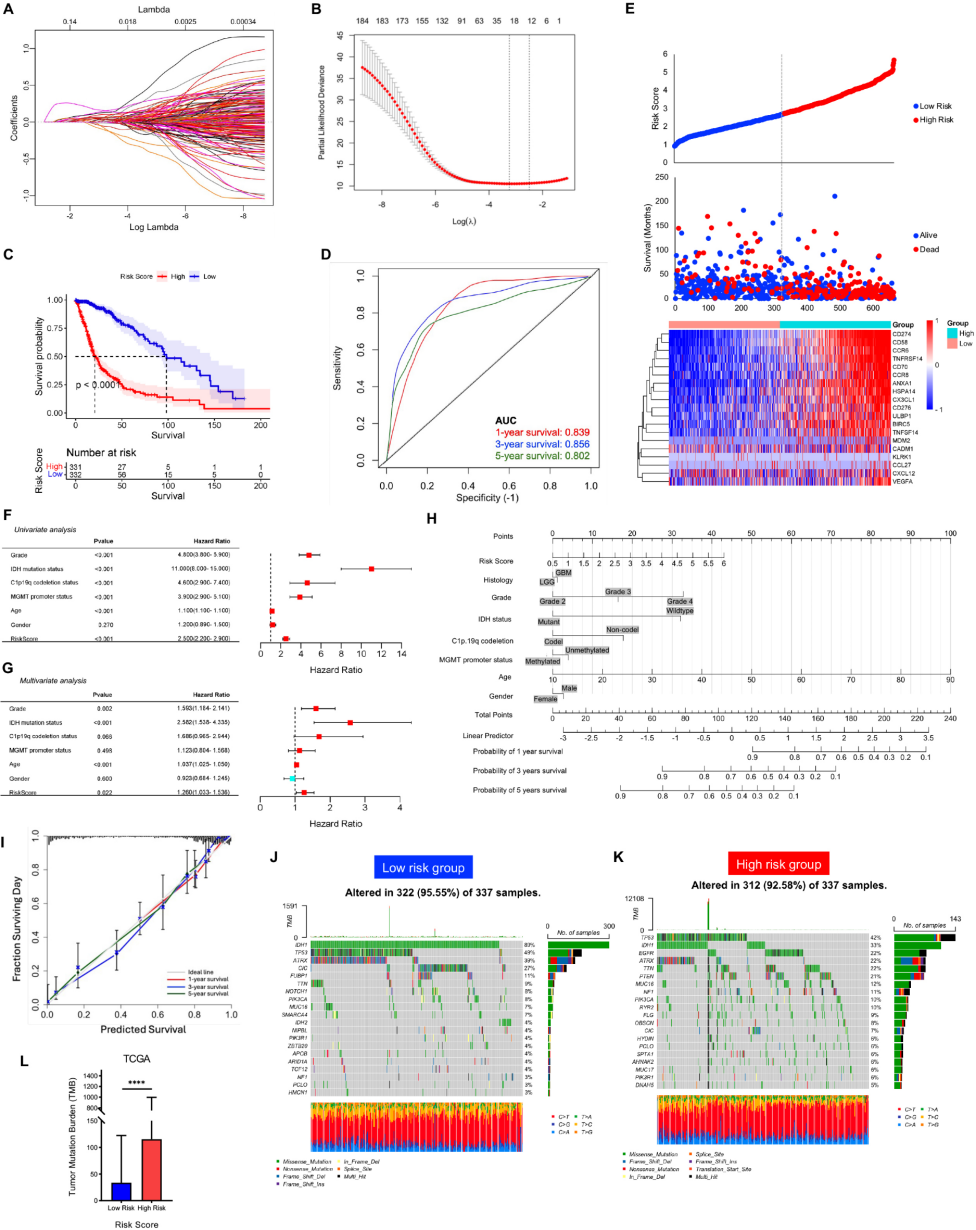
MAN1C1-based immune prognostic-risk model and tumor mutation burden. (**A**,**B**) The least absolute shrinkage and selection operator was utilized to construct a MAN1C1-based immune-prognostic risk model. (**C**) Kaplan–Meier analysis between low-risk and high-risk groups. (**D**) ROC curves were used to compare the predictive efficacy of the MAN1C1-based immune-prognostic risk model based on 1-, 3- and 5-year survival. (**E**) The distribution of risk score of the glioma patients, survival status of the patients based on the risk score, and expression of the 19 genes between the high-risk and the low-risk groups. (**F,G**) Univariate and multivariate Cox proportional hazards regression analysis of variables influencing overall patient survival in the high-risk and the low-risk groups. (**H**) Nomogram for predicting overall survival. (**I**)**.** The calibration curve of the nomogram model. (**K**,**L**) Mutation landscape high-risk and low-risk groups. (**K**) Comparison of the tumor mutation burden between the landscape high-risk and the low-risk groups.

To create a MAN1C1-IPS risk model, 249 immunomodulators, chemokines, and genes related to the cancer-immunity cycle were obtained from the TISIDB and TIP databases. We used Pearson’s correlation analysis on the TCGA dataset to determine the genes associated with MAN1C1 expression. In total, 215 genes were revealed to be associated with MAN1C1 expression (Supplementary Table 3). We used univariate Cox regression to determine the prognostic relevance of these genes. We found that 186 genes had significant prognostic relevance in patients with glioma (Supplementary Table 4). These genes were then subjected to LASSO regression to create a model that fit across multiple lambda values (Fig. 6A) and was assessed using partial likelihood deviance (Fig. 6B). The coefficients of the chosen gene model (Supplementary Table 5) were used to calculate the risk scores in the TCGA dataset. The patients were then grouped as low- or high-risk based on their median risk score. Survival analysis revealed that patients in the low-risk group had longer OS than those in the high-risk group (Fig. 6C). Furthermore, the ROC curve demonstrated that the model could predict 1-year, 3-year, and 5-year OS (Fig. 6D). Patients with higher risk scores had a poor prognosis (Fig. 6E). Furthermore, univariate and multivariate analyses demonstrated that a higher risk score was associated with shorter OS (Fig. 6F,G). These data indicate that the risk score is an independent prognostic factor for OS in glioma patients. Subsequently, a multivariate Cox regression model was built using the independent prognostic factors for OS. In the TCGA dataset, the risk score ranged from 0 to 100 (Fig. 6H). The C-index value used to construct the nomogram was 0.866. Excellent agreement was found in the calibration plot between the observed and projected 1-, 3-, and 5-year OS likelihoods (Fig. 6I). This indicates that the signature is accurate for predicting OS. Finally, we investigated the differences in mutation levels between the low- and high-risk subgroups. The low-risk group had increased mutation levels of IDH1, TP53, ATRX, and CIC (Fig. 6J). Conversely, the high-risk group had a greater frequency of EGFR, PTEN, PIK3CA, and NF1 mutations (Fig. 6K). Our results show that various mutations may exist in gliomas based on their risk scores.

### MAN1C1 expression indicates the TME phenotype in gliomas

**Fig. 7:**
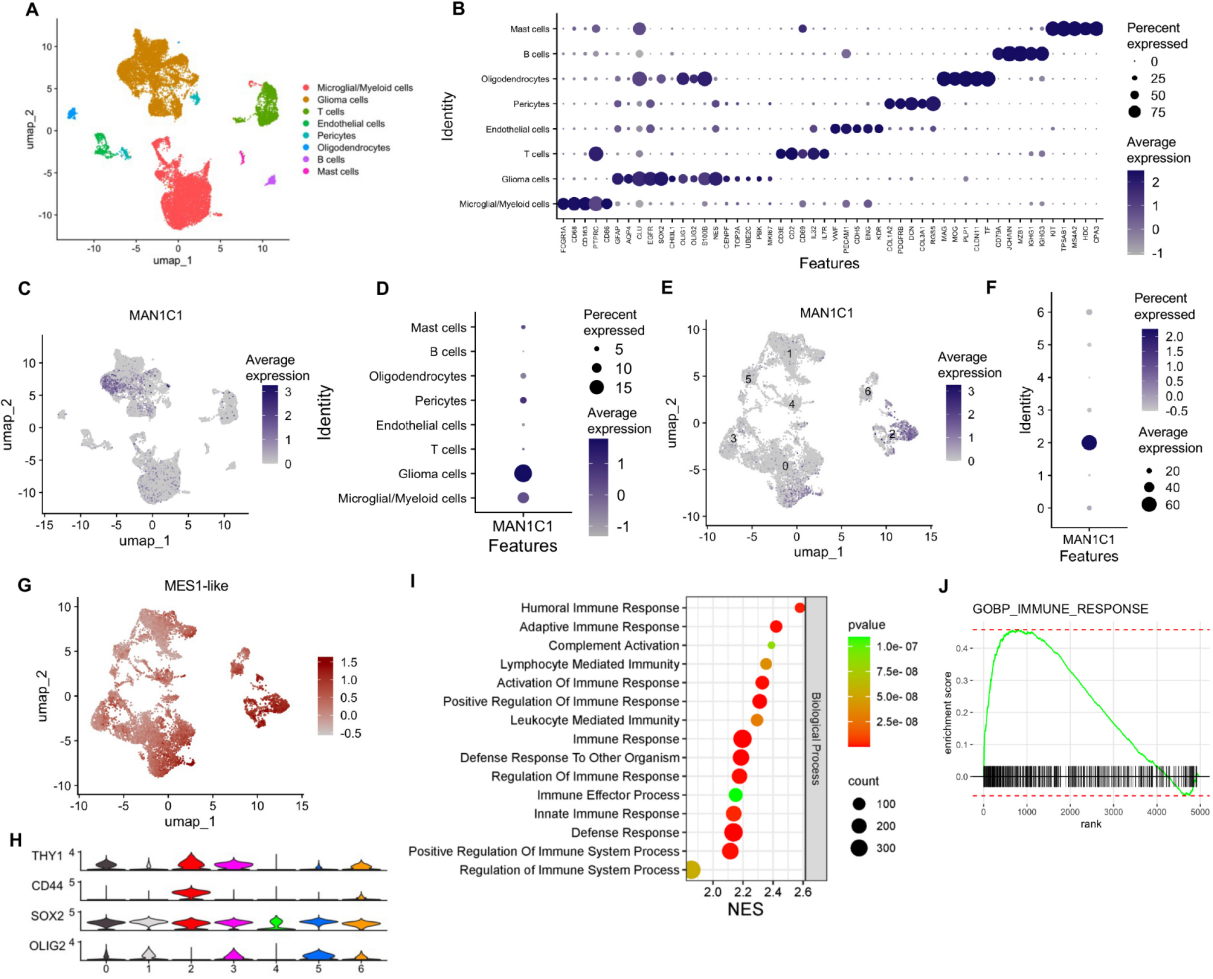
MAN1C1 is elevated in mesenchymal gliomas based on scRNA-sequencing analysis. (**A**) DimPlot of different cell clusters. (**B**) DotPlot of marker genes and grouping it by each cluster. (**C**) FeaturePlot of cell clusters expressing MAN1C1. (**D**) DotPlot of MAN1C1 expression in different cell clusters. (**E**) FeaturePlot of glioma cell clusters expressing MAN1C1. (**F**) DotPlot of MAN1C1 expression in different glioma cell clusters. (**G**) FeaturePlot of MES1-like phenotype enrichment in the glioma cell cluster. (**H**) Stacked violin plot of stemness markers in the glioma cell clusters. (**I**) Dot plot of the functional enrichment result of DEGs in the glioma cluster 2 cells. (**J) **plotEnrichment of immune response gene set in the glioma cluster 2 cells.

To better understand the role of MAN1C1 in gliomas and the TME, we analyzed the scRNA-seq dataset (GSE182109) from three primary GBM samples. Unsupervised analyses revealed eight cell clusters representing stromal, immune, and glioma cells (Fig. 7A). Glioma cells expressed GFAP, AQP4, CLU, EGFR, SOX2, OLIG1, OLIG2, S100B, NES, and CH3L1 (Fig. 7B). CENPF, TOP2A, UBE2C, PBK, and MKI67 were strongly expressed in glioma clusters, indicating the presence of progenitor cells. Moreover, immune cells, including myeloid/microglia (FCGR1A, CD68, CD163, PTPRC, and CD86), T cells (CD3E, CD2, CD69, IL32, and IL7R), and B cells (CD79A, JCHAIN, MZB1, IGHG1, and IGHG3), were also identified. The stromal cells present included pericytes (COL1A2, PDGFRB, DCN, COL3A1, RGS5), endothelial cells (VWF, PECAM1, CDH5, ENG, KDR), and mast cells (KIT, TPSAB1, MS4A2, HDC, CPA3). Moreover, MAN1C1 was substantially expressed in a portion of the glioma cell cluster (Fig. 7B,C). We subsequently grouped the glioma cells to further classify cells expressing MAN1C1 (Supplementary Fig. S6A). MAN1C1 was overexpressed in glioma cluster 2 cells exhibiting a MES1-like cell state based on their module scores (Fig. 7E–G). Other glioma cell clusters exhibited MES2-, AC-, OPC-, and NPC1/2-like cell states (Supplementary Fig. S6B–F). A fraction of the glioma cell clusters were also highly proliferative, as evidenced by the G1/S and G2/M scores (Supplementary Fig. S6G,H).

To validate the presence of GSCs, we examined the expression profiles of proneural (SOX2, OLIG2) and MES stem cell markers (CD44, THY1) (Fig. 7H). Both THY1 and CD44 were significantly expressed in cluster 2 cells. Moreover, SOX2 was strongly expressed in all cell clusters, while OLIG2 was significantly expressed in all glioma cell clusters, except for 2 and 4. The expression levels of N-glycosylation-related genes differed in glioma cell clusters. The expression of N-acetylglucosaminyltransferases (GALNT2, GALNT10, GALNT11, and GALNT2), galactosyltransferases (B4GALT1 and B4GALT5), mannosidase (MAN1A2), fucosyltransferase (FUT9), and sialidase (NEU1) increased in MAN1C1-expressing cells (Supplementary Fig. S6I). These genes are critical for N-glycan maturation and branching. Additionally, glioma cluster 2 cells expressed selectins (LGALS1 and LGALS3), integrins (ITGB1), and glycosylation targets (VIM, TIMP1, HIF1A, and CD44) (Supplementary Fig. S6J–L). Next, we used FGSEA to identify the enriched biological processes in glioma cluster 2 cells. The results demonstrated that immunological and inflammatory responses were concentrated in glioma cluster 2 cells (Fig. 6K,L). These findings suggest that MAN1C1-expressing cells may be involved in the immune response, thereby helping shape the TME.

### Secreted signaling pathways allow communication between MAN1C1-expressing glioma cells and other cells in the TME

**Fig. 8:**
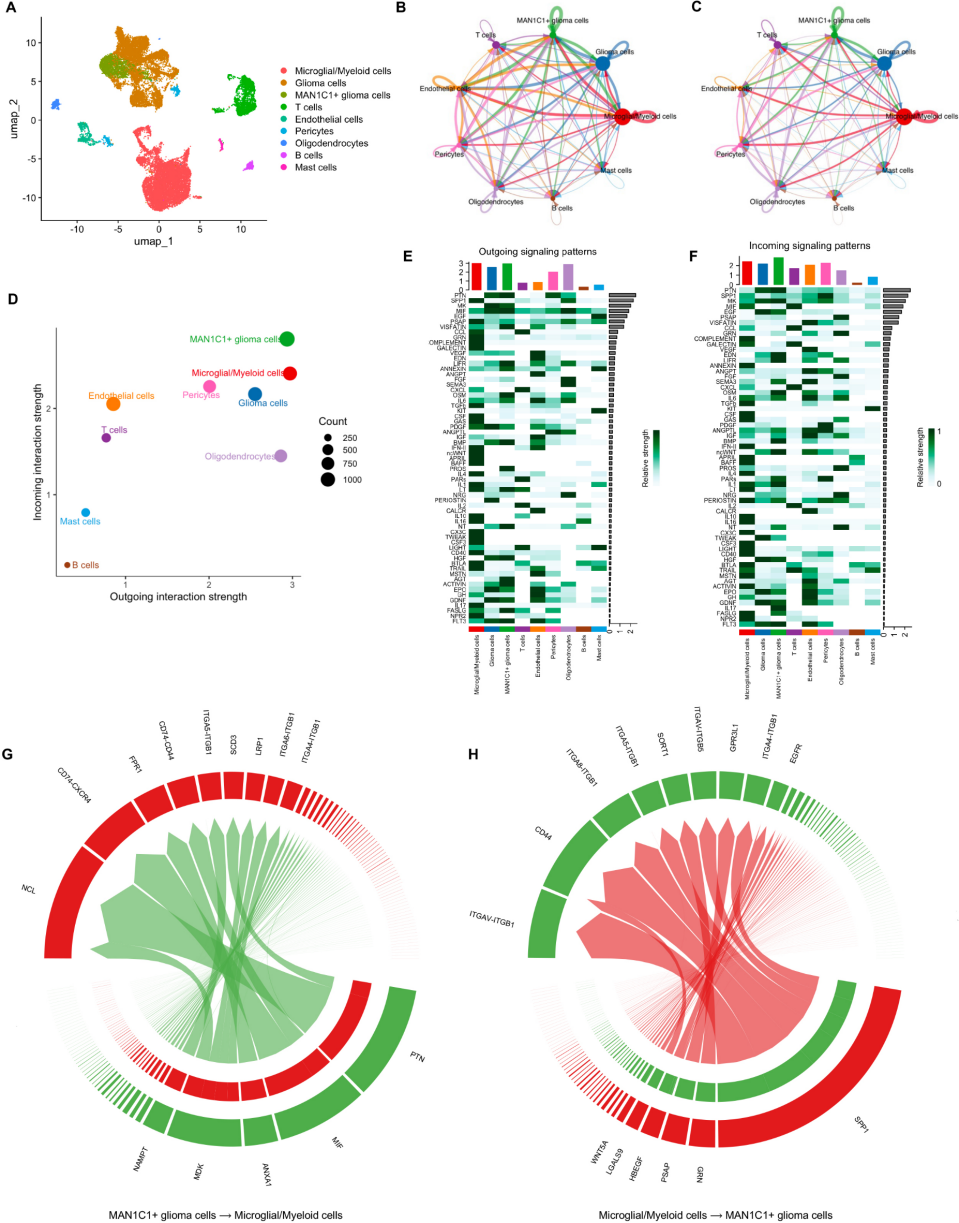
Secreted-signaling pathways allow communication between MAN1C1-expressing gliomas and other cells in the TME. (**A**) Clustering and annotation of MAN1C1-expressing cells and other cell populations. (**B**,**C**) The circle interaction plots show the count (**B**) and weight (**C**) of inferred intercellular communication network analysis of MAN1C1-expressing glioma cells with other cell clusters. (**D**) Magnified view of each cell group’s total incoming or outgoing strength. Circle size represents the signaling network counts. Different colors represent different groups of cells. The X-axis means total received signal strength; the Y-axis means total sent signal strength. (**E**,**F**) Contribution of the pathways identified to the (**E**) outgoing and (**F**) incoming signals among the different cell types. (**G**,**H**) Chord diagrams representing the signaling between MAN1C1-expressing glioma cells and microglial/myeloid cell cluster.

We used scRNA-seq data to identify potential ligands and receptors for cell-cell interactions between MAN1C1-expressing cells and TME. First, we isolated MAN1C1-expressing glioma cells from the remaining glioma cell clusters (Fig. 8A). The circle interaction plots depict the count and weight of the inferred high intercellular communication network between MAN1C1-expressing glioma cells and other cell clusters (Fig. 8B,C). An increase in contact strength indicates a strong interaction between MAN1C1-expressing glioma cells, other glioma cells, and myeloid/microglial cells (Fig. 8D). The outgoing signaling pattern in MAN1C1-expressing glioma cells showed that PTN, MK, MIF, VISFATIN, LIF, LIFR, ANNEXIN, OSM, IL6, PDGF, BMP, PROS, LT, NT, HGF, ACTIVIN, AGT, GDNF, FASNG, and FLT3 signaling pathways were significantly activated. In contrast, incoming signaling patterns in MAN1C1-expressing glioma cells stimulated the PTN, SPP1, MK, EGF, EDN, LIFR, OSM, IL6, ANGPLT, IGF, BPM, ncWNT, PARs, IL1, PERIOSTIN, NT, TWEAK, HGF, EPO, GDNF, IL17, FASLG, and FLT3 signaling pathways (Fig. 8F). As myeloid/ microglial cells had the strongest interaction with MAN1C1-expressing glioma cells (Fig. 8D), we investigated the ligands and receptors involved. Secreted signals in MAN1C1-expressing glioma cells, such as PTN, MIF, ANXA1, MDK, and NAMPT, allowed MAN1C1 to interact with myeloid/microglial cells (Fig. 8G). Myeloid/microglial cells can communicate with MAN1C1-expressing glioma cells via signals produced (SPP1, GRN, PSAP, HBEGF, LGALS9, WNT5A) (Fig. 8H). In MAN1C1-expressing glioma cells, the presence of highly glycosylated receptors, such as CD44 and integrins (ITGB1, ITGAV, ITGA8, ITGAB1) facilitates this interaction. These results imply that MAN1C1 expression might be essential for shaping the surface proteins of glioma cells, enabling them to communicate with other cells and the TME.

### MAN1C1 is correlated with immune checkpoints and the ICB response

While ICB combination treatment performs well in preclinical glioma models, further studies are needed to determine its effectiveness in GBM patients. Using the TIDE algorithm, we examined the association between the ICB response and MAN1C1 expression in the TCGA_GBM dataset. Patients who expressed low levels of MAN1C1 were 50% more likely to respond to ICB therapy than those who expressed high levels of MAN1C1 (48%) although not significant (Supplementary Fig. S7A,B). T-cell dysfunction was much greater in patients with low MAN1C1 expression, while T-cell exclusion was significantly greater in patients with MAN1C1^High^ expression (Supplementary Fig. S7C,D). This finding suggests that the T cells in the TME are capable of detecting and destroying cancer cells efficiently. The exclusion could be due to physical barriers such as a dense extracellular matrix or immunosuppressive substances that make the TME unsuitable for T-cell invasion. TIDE analysis revealed that GBM patients with MAN1C1^High^ expression had high infiltration of MDSCs and M2 TAMs, whereas patients with low MAN1C1 expression had much greater infiltration of CAFs (Supplementary Fig. S7E-F). We then investigated the link between MAN1C1 expression and the well-studied checkpoints. MAN1C1 was strongly correlated with PD-L1, PD-L2, and TIM-3 in the TCGA dataset. Overall, these findings indicate that GBM patients with MAN1C1^High^ expression may be unable to respond to ICB treatment, particularly to anti-PD-1 therapy.

## Discussion

Abnormal protein glycosylation has been observed to have a role in the progression and metastasis of many cancers in recent years^12,13^. In GBM tissues, mannosidase and glucosidase enzymes cleave mannose and glucose residues from Man9 − 4GlcNAc2 oligosaccharide precursors, converting high mannose type N-glycans to complex type N-glycans^21^. Peripheral modifications, fucosylation, and sialylation of N-glycan structures promote tumor progression, invasion, migration, self-renewal immunosuppression, and therapeutic resistance in gliomas^7^. During the early stages of glycosylation, mannosidases remove mannose residues from polysaccharides and complex glycoconjugates^11^. This step is essential because it provides a substrate for various glycosyltransferases, facilitating the synthesis of high-mannose, hybrid, or complex/branched N-glycans^19,20^. Mannosidases have been studied in various cancers, however, their role in glioma is very limited^11^.

In this study, we performed a comprehensive in-silico analysis of various public datasets to elucidate the significance and underlying mechanisms of N-glycosylation-related genes in GSC phenotypes and GBM malignancy. We demonstrated that α-1,2-mannosidase MAN1C1 is significantly expressed in GSCs. In various cancers, MAN1C1 acts as an oncogene^26,27^ or tumor suppressor^24,25,28^. Hence, we postulate that its role depends on cellular context. Meanwhile, MAN1A2, GCNT2, MGAT3, MGAT4A, and FUT9 were also found to be elevated in GSCs. Although there are no reports of these genes in gliomas and GSCs, they have been identified in the progression of other cancers^33–36^. These findings provide an important starting point for additional validations to determine the relevance of glycosylation genes in gliomas as a diagnostic and therapeutic target.

We also found that elevated MAN1C1 expression predicts worse outcomes in patients with glioma, indicating that MAN1C1 could be a potential biomarker for predicting glioma patient survival. This result has also been demonstrated in various cancers^16,25,26,37^. We also showed that MAN1C1 was elevated in the GBM-MES subtype. This result agrees with the findings of a previous study^11^. The MES subtype has the worst prognosis among all GBM subtypes. It is characterized by elevated expression of NF-κB pathway and TNF superfamily genes, along with traits such as necrosis, angiogenesis, strong immune and inflammatory responses, and loss of tumor suppressor genes^38–40^. Using the Ivy-GAP dataset and spatial transcriptomic data, we showed that MAN1C1 is highly expressed in the perinecrotic region of GBM. Accordingly, microvascular proliferative and perinecrotic/hypoxic regions are common niches of the GBM-MES subtype^39,41^. Co-expression analysis revealed that MAN1C1 is significantly associated with MES, hypoxia, and perivascular marker expression. The hypoxia gene HIF1A is known to enhance CD44 expression in GBM^42^. The intracellular domain of CD44 binds to HIF-2α (but not HIF-1α) and promotes HIF target gene activation to cause hypoxia-induced stemness in gliomas^43^. Moreover, GBM-MES cells have a dense glycocalyx, which promotes integrin-mediated mechanosignaling and an invasive stem cell-like phenotype^44^. Recently, MAN1A1 (MAN1C1 paralogue) was reported to be downregulated in CD133 + glioma stem cells, suggesting that high-mannose type N-glycan could be an enrichment signal for proneural GSC^45^. We hypothesized that MAN1C1 expression may influence the aggressive characteristics of the GBM-MES subtype.

Through comprehensive functional enrichment analysis, we showed that MAN1C1 is associated with various processes (immune and inflammatory responses, hypoxia, apoptosis, angiogenesis, and cell adhesion), and pathways (IL6/JAK-STAT3, IL2/STAT5, and TNF-α signaling pathways via NF-kB and TGF-β signaling). The NF-κB and STAT3 signaling pathways are involved in increased cytokine production, invasion, cell cycle progression, apoptosis, and angiogenesis^46,47^. In a previous report, it was found that the TNF-α, IL1B, and TGF-β signaling pathways induced by radiation negatively regulate MAN1C1 expression^48^. MAN1C1 downregulation by TNF-α increases the abundance of high-mannose glycans and triggers a proinflammatory effect. This can be reversed by activating the PPAR signaling pathway, which enhances MAN1C1 expression and supports the anti-inflammatory pathway^49^. Further research is needed to investigate how these pathways regulate MAN1C1, and how this affects the aggressive features of GBM.

The complex heterogeneity of GBM cells is facilitated by the local inflammatory TME, which primarily promotes tumor aggressiveness and resistance to treatment^50^. We found that MAN1C1 expression was associated with increased neutrophil, MO/M2 macrophages, and CD4 + T-cell infiltration, but not CD8 + T-cell infiltration. Changes in glycosylation affect cellular infiltration, tumor invasion, antigen-antibody interactions, oncogenic signal transduction, ligand-receptor interactions, cell motility, and cell-matrix interactions^15,51^. We also showed that MAN1C1 expression is correlated with the expression of various ligands, receptors, and immunomodulators. The aberrant glycosylation of cell surface receptors and secreted proteins in cancers alters their interaction with immune cells as well as their proliferation and survival^52^. We believe that elevated MAN1C1 expression may affect the glycan structure and surface proteins of gliomas, influencing immune cell infiltration or evasion. Therefore, extensive in vitro and in vivo experiments are required to support this premise.

A MAN1C1-IPS was constructed and validated using the TCGA dataset, resulting in a considerably more accurate prediction of glioma prognosis than clinicopathological variables. A high-risk score indicates enrichment of well-known aggressive characteristics, indicating that a high-risk score may predict poor outcomes in glioma patients. The low-risk group had a better prognosis, which was consistent across clinical variables. These findings suggest that MAN1C1-IPS may be a beneficial prognostic indicator of glioma outcomes. Additionally, the MAN1C1-IPS risk score is linked to somatic changes, malignant characteristics, and clinical features of gliomas. Somatic mutations in the low-risk group included more IDH1, TP53, ATRX, and CIC mutations. Compared to wild-type IDH, IDH-mutant is an independent predictor of better OS and progression-free survival in glioma patients^53^. The high-risk group included patients with mutations in genes, such as EGFR, PTEN, and NF1. Glioma-related genomic changes may lead to immune cell infiltration and chemokine overexpression. Mutations in NF1, PTEN, and EGFR are related to enhanced recruitment of tumor-associated macrophages (TAMs)^54^. Moreover, increased PTEN mutations in gliomas are associated with immunosuppressive expression signatures^55^. Further research is needed to understand the correlation between mutation status and MAN1C1 expression in patients with malignant gliomas.

We employed scRNA-seq to further investigate the role of MAN1C1 in gliomas and TME. We found that MAN1C1 was highly expressed in gliomas with MES1-like (hypoxia-independent) gene signatures. The MES1-like signature is characterized by high expression of CD44 ^56^. Spatial and scRNA-seq analyses revealed that MAN1C1 was co-expressed with CD44. Specifically, CD44 was found in a perinecrotic, hypoxic niche with HIF-1α- and HIF-2α-positive glioma cells, as well as a perivascular, highly oxygenated niche with pseudo-hypoxic, HIF-2α-positive, stem-like cells^43^. Our enrichment analysis demonstrated that the MAN1C1-expressing glioma cell cluster is associated with immune- and inflammatory-related genes. Accordingly, immunological and inflammatory activities may enhance the formation of the MES signature, as evidenced by the link between immune-related gene expression and MES GBM^57^. This finding suggests that MAN1C1 expression may be involved in shaping MES signatures in GBM; hence, further investigation is required.

According to cell-cell interaction analysis, MAN1C1-expressing glioma cells strongly interact with myeloid/microglial cell clusters. The production of signaling factors (PNT, MIF, ANXA1, and MDK) in MAN1C1-expressing glioma cells enables communication with microglial/myeloid cell clusters. These signaling factors have been shown to allow immune escape by increasing the anti-inflammatory TME^58–61^. MAN1C1-expressing cells exhibited high levels of CD44. Increased expression of CD44 is correlated with poor prognosis in patients with GBM^62^. High glycosylation of CD44 affects its binding to HA, Siglec-15, fibronectin, TM4SF5, PRG4, FGF2, collagen, and podoplanin, and activates or inhibits various signaling pathways^63^. We found that MAN1C1-expressing glioma cells interact with microglial/myeloid cells via the SPP1-CD44 signaling pathway. This finding supports the results of a previous study^64^. OPN/SPP1 has cytokine, chemokine, and signal transduction capabilities through modular structural motifs that interact with integrins and CD44-variant receptors^65^. Notably, complex-type glycans post-translationally modify the CD44 extracellular structure at most glycosites, whereas high-mannose-type N-glycans occupy the N100 glycosyl site^66^. Five potential N-linked glycosylation sites on CD44 are required for CD44-mediated adhesion to HA in human cell lines^67^. The CD44-HA interaction has been found to promote immune evasion by inhibiting Fas expression during Fas/Fas ligand T cell-mediated cytotoxicity in lung cancer cells^68^. Also, TAMs promote the interaction between HA and CD44 through HAS2, which enhances stemness through PI3K-4EBP1-SOX2 signaling pathway activation in head and neck CSCs^69^. Further in vitro and in vivo experiments are needed to determine the glycosylation patterns that facilitate the aggressive characteristics of GSCs.

The TIDE algorithm can predict whether a patient will respond to ICB therapy. Our findings indicate that patients with low MAN1C1 expression levels respond to ICB therapy. Moreover, T-cell dysfunction was greater in patients with low MAN1C1 expression, whereas T-cell exclusion was significantly greater in patients with h MAN1C1^High^ expression. We assume that T cells in the TME are fully functional and capable of detecting and destroying cancer cells. However, the exclusion could be due to physical barriers, such as a dense extracellular matrix or immunosuppressive substances that make the TME unsuitable for T-cell invasion. N-glycosylation is also essential for some immunosuppressive receptor-ligand interactions, including interactions between PD-1/PD-L1, B7-1/PD-L1, and PD-1/PD-L2 ^70^. Similarly, TCR signaling can control CTLA-4 N-glycan branching, resulting in increased surface retention, which suppresses T-cell activity and drives immune evasion^71^. The loss of TNF signaling components has been linked to immunological evasion by CD8 + T cells and NK cell-mediated killing^72^. Moreover, in PD-1 inhibitor non-responders, significant enrichment of PTEN mutations is associated with immunosuppressive expression patterns^55^. Our findings imply that MAN1C1 expression can be used to predict glioma patient response to ICB treatment. Thus, altering glycosylation may be a viable strategy for increasing ICB responsiveness and overcoming current barriers to GBM therapy.

In summary, we found that the α-1,2-mannosidase MAN1C1 was elevated in GSCs and was linked to the clinical, pathological, and molecular characteristics of gliomas. A combined transcriptomics study of bulk, spatial, and scRNA-seq data revealed that MAN1C1 is related to MES characteristics in GBM, contributing to a poor overall prognosis in glioma patients. Enrichment analysis revealed that increased MAN1C1 levels are associated with immunological and inflammatory-related genes, perhaps leading to infiltration of immune cells and dysregulated immune responses in gliomas. Taken together, the findings show that MAN1C1 is a predictive biomarker in glioblastoma and could be a new target for developing immunotherapies for GBM.

### Methods

#### Selection of bulk transcriptomic samples, preprocessing, and DGE analysis

The list of N-glycosylation-related genes, regulators, and targets (Supplementary Table 1) was obtained from a previous study^73^. RNA-seq data (GSE4536) of GSCs in differentiated (serum) and stem cell (NBE) cultures were obtained from the NCBI GEO Dataset^74^. Integrated differential expression and pathway analysis (iDEPver.1.1; (http://bioinformatics.sdstate.edu/idep11/) was used to normalize and transform (log2 CPM + 1) the bulk RNA-seq data. A |log2FC| ≥0.5 and a p value < 0.05 were considered indicative of the biological significance of the differentially expressed genes (DEGs). The clinical and expression data of glioma patients (TCGA_GBMLGG, TCGA_GBM, CGGA, Gravendeel, and Ivy-GAP) were obtained from the Gliovis database (http://gliovis.bioinfo.cnio.es).

### Expression and survival analysis

The R ‘ggplot’ package was utilized to evaluate the expression profiles and clinical data in the TCGA and CGGA datasets. The R packages ‘survminer’ and ‘survival’ were used to carry out univariate and multivariate Cox proportional regression analyses. The R ‘caret’ tool was used to divide the datasets into training and test cohorts. The R ‘survival’ and ‘survivalROC’ packages were used to construct Kaplan-Meier (KM) and receiver operating characteristics (ROC) curves, respectively.

### Functional enrichment analysis

The Gene Ontology (GO) in terms of biological processes, cellular components, molecular functions, and KEGG pathways were annotated on the Database for Annotation, Visualization, and Integrated Discovery (DAVID) website (https://david.ncifcrf.gov/). The related hallmark gene sets were analyzed using the Gene Set Enrichment Analysis (GSEA) program (version 4.2.2).

### Construction of the co-expression network and identification of hub gene modules

The ‘WGCNA’ R package was used to construct a co-expression network of genes from the TCGA dataset. Clustering analysis was performed to remove outlier samples to ensure network dependability. To optimize the scale-free topology, a model fitting index (R2 > 0.9) and a soft threshold power of 7 was employed. A power function was used to generate the adjacency matrix of topology similarity, which was then translated into a topological overlap matrix. The matching dissimilarity (1-TOM) was calculated using the distance to hierarchically cluster genes as a reference. This enabled the dynamic tree-cut technique to identify modules and generate a dendrogram. The module eigengene (ME) and signedKME functions were used to calculate the module membership (MM). Metascape (http://Metascape.org) was used to perform functional enrichment analysis on the gene hubs in each ME.

### Immune cell infiltration analysis

The ESTIMATE algorithm was used to predict tumor purity in the TCGA dataset based on stromal, immunological, and ESTIMATE scores (https://bioinformatics.mdanderson.org/estimate/). The TIMER 2.0 website (http://timer.comp-genomics.org/timer/) was used to determine the abundance of tumor infiltrates. The Tracking Tumor Immunophenotype (TIP) website (https://biocc.hrbmu.edu.cn/TIP/index.jsp) was utilized to examine the seven steps of the cancer immune cycle. Tumor-immune system interactions were analyzed using the TISIDB website (http://cis.hku.hk/TISIDB/).

### Construction of a MAN1C1 immune prognostic risk score model

In the TCGA dataset, univariate Cox proportional regression analysis was used to assess the relationships between the genes linked to OS. We used the R ‘glmnet’ package to develop a prognostic risk model by applying the LASSO method to genes with prognostic relevance. Finally, each patient’s risk score in the TCGA datasets was calculated using the following formula:

$$\:\text{R}\text{i}\text{s}\text{k}\:\text{S}\text{c}\text{o}\text{r}\text{e}\:=\:\sum\:_{\text{i}=0}^{\text{n}}{\text{C}\text{o}\text{e}\text{f}}_{\text{i}}\text{*}{\text{x}}_{\text{i}}$$

where Coef_i_ is the coefficient and x_i_ is the expression value of each selected gene. Patients were divided into low- and high-risk groups according to the median risk score. Thereafter, univariate and multivariate Cox regression analyses and KM survival and ROC curve analyses were performed. Finally, a nomogram was constructed using the R ‘rms’ package to determine whether the risk scores could improve the predictive performance of the model.

### Tumor mutation burden (TMB)

Mutation data for glioma patients were obtained from the R ‘TCGAmutations’ package, and data analysis was performed with the R ‘maftools’ package. Using TCGA somatic variations, the raw mutation count for TMB analysis was calculated.

### Analysis of tumor immune dysfunction and exclusion (TIDE)

The normalized TCGA_GBM transcriptomic data were uploaded to the TIDE website (http://tide.dfci.harvard.edu) to calculate the TIDE score and immune checkpoint blockade (ICB) response in GBM patients.

### Spatial transcriptomics analysis

The glioblastoma spatial transcriptomic data was downloaded from 10x Genomics (https://www.10xgenomics.com/datasets/human-brain-cancer-11-mm-capture-area-ffpe-2-standard) and analyzed in the R ‘Seurat’ package. The data were normalized using the SCTranform function. PCA and UMAP were then utilized for dimension reduction, and the first 30 PCs were clustered at standard resolution. The SpatialFeaturePlot function was used to depict features related to gene expression. Spatially variable features were identified using the FindMarkers function. The DEGs were annotated on the DAVID website and visualized using the R ‘ggplot’ package.

#### scRNA-seq analysis

From the GSE182109 dataset^75^, the scRNA-seq data of three recently diagnosed GBM samples (GSM5518633, GSM5518634, GSM5518636) were analyzed using the ‘Seurat’ R package. Low-quality cells with total RNA features (< 200) and mitochondrial RNA (> 20%) were removed before the scRNA-seq samples were merged using the SCTransform integration algorithm. The data were normalized with the NormalizeData function, and the FindVariableFeatures method was used to identify the top 2000 highly variable genes. The RunPCA program was used to perform principal component analysis, and the top 20 principal components were chosen for cell clustering analysis. The FindAllMarkers program was used to identify DEGs in each cluster, with thresholds of FDR < 0.05 and |Log2 (FC)| > 0.25. To perform cell type annotation, marker genes in every cluster were manually added to CellMarker2.0 (http://biobigdata.hrbmu.edu.cn/CellMarker/index.html). The AddModuleScore method was used to identify gene scores for cellular states (MES1/2, AC, OPC, and NPC1/2-like, and G1/S and G2/M)^56^. The FGSEA function was used to perform enrichment analysis. The plotEnrichment, DotPlot, DimPlot, FeaturePlot, and DOHeatmap functions were used to visualize the results.

### Cell–cell interaction analysis

Cell–cell interactions between clusters from the scRNA-seq data can be identified and visualized using the R ‘CellChat’ package. This package considers the structural composition of cofactor molecules and ligand-receptor interactions and includes supporting evidence for each signal.

#### Cell culture

Normal human astrocyte (NHA) and non-stem cell GBM lines (A172, A1207, U87MG, LN229) were purchased from the American Type Culture Collection (ATCC). The University of Texas MD Anderson Cancer Center provided the GSC lines (GSC11, GSC23, GSC20, and GSC267)^29^. NHA was cultured in astrocyte medium (ScienCell Research Laboratories, USA) supplemented with 10% FBS (Gibco, USA), 1% penicillin/streptomycin (P/S; Welgene, South Korea), and 1% Astrocyte growth supplement (AGS; ScienCell Research Laboratories, USA). Non-stem cell GBM cell lines were cultured in DMEM/F12 (Welgene, South Korea) supplemented with 1% P/S and 10% FBS. GSCs were grown in neurobasal (NBE) medium supplemented with DMEM/F12, 20 ng/ml bFGF and EGF (R&D Systems, USA), 2% B27 (Gibco, USA), and 1% P/S. All cells were maintained at 37 °C with 5% CO_2_.

#### Quantitative Reverse Transcription‒PCR (qRT‒PCR) analysis

Total RNA was extracted with the GeneAll^®^ RiboEx™ reagent and purified using the GeneAll^®^ Hybrid-R™ kit according to the manufacturer’s instructions (GeneALL Biotechnology, South Korea). A RevertAid™ First Strand cDNA Synthesis Kit (Thermo Fisher Scientific, USA) was used to convert total RNA (500 ng) to cDNA. RT‒qPCR was carried out using a qTOWER3 Real-Time PCR thermocycler (Analytik-Jena, Germany) with Tli RNaseH Plus TB Green^®^ Premix Ex Taq™ (Takara, South Korea). The 2^−ΔΔCt^ method was used to examine relative changes in gene expression, which were then normalized against the expression level of the 18 S housekeeping gene. The experiment was independently replicated three times. The sequences of the primers used in this study are listed in Supplementary Table 2.

### Statistical analysis

All the data analyses were carried out using GraphPad Prism version 8.3 (GraphPad Software, San Diego, CA, USA) and R version 4.3.2. Pearson’s R correlation was used for all correlation analyses. The statistical significance of differences between groups was established using two-tailed t-tests and one-way ANOVA. The results were then evaluated with Tukey’s multiple comparison tests. All the data are reported as the means ± standard errors (SEs). A p value ≤ 0.05 was considered to indicate statistical significance (*p* < 0.05 = *; <0.01 = **; <0.001 = ***; <0.0001 = ****; ns = not significant).

## Electronic supplementary material

Below is the link to the electronic supplementary material.

Supplementary Material 1

Supplementary Material 2

Supplementary Material 3

## Data Availability

The datasets presented in this study were accessed in online repositories. GSC RNA-seq data (GSE4536), and Single-cell RNA-seq data (GSE182109) of three recently diagnosed GBM samples (GSM5518633, GSM5518634, GSM5518636) were obtained from NCBI GEO (https://www.ncbi.nlm.nih.gov/geo/). Clinical and expression data of glioma patients were obtained from the Gliovis database (http://gliovis.bioinfo.cnio.es). Glioblastoma spatial transcriptomic data was downloaded from 10x Genomics (https://www.10xgenomics.com/datasets/human-brain-cancer-11-mm-capture-area-ffpe-2-standard).
