# Supplementary material for: Elevated α-1,2-mannosidase MAN1C1 in glioma stem cells and its implications for immunological changes and prognosis in glioma patients
Source: Sci Rep. 2024 Sep 27;14:22159. doi: 10.1038/s41598-024-72901-2 (PMC11436702; doi:10.1038/s41598-024-72901-2)
Supplement: Supplementary file 2 — Supplementary Material 2 [file 41598_2024_72901_MOESM2_ESM.docx]

**Supplementary Figure Legends**

**Supplementary Figure 1.** Protein glycosylation pathway and mannosidase mRNA expression. **(A)** Overview of protein N-glycan biosynthesis pathway. **(B-D)** qRT-PCR analysis of the MAN1A1 (B), MAN1A2 (C) and MAN1B1 (D) basal mRNA levels in GBM cell lines (A172, A1207, U87MG, LN229), GSC lines (GSC11, GSC20, GSC23, GSC267), and NHA cells.

**Supplementary Figure 2.** MAN1C1 is highly expressed in high-grade gliomas and correlate to poor patient outcomes in the CGGA dataset. **(A)** MAN1C1 mRNA expression and clinical features in the CGGA dataset (n = 1013); **(B)** Raincloud plot of MAN1C1 expression in GBM and LGG patients; **(C-D)** Univariate and multivariate Cox proportional hazards regression analysis of variables influencing overall patient survival in the TCGA dataset. **(E-F)** Kaplan-Meier analysis of glioma patients with MAN1C1 High/Low expression in (E) training and (F) validation set in the TCGA dataset. **(G-H)** ROC curve and the risk score distribution stratified by MAN1C1 High/Low expression in (G) training and (H) validation set in the TCGA dataset.

**Supplementary Figure 3.** MAN1C1 is correlated to poor prognosis in GBM patients. **(A-C)** Kaplan-Meier analysis of GBM patients with MAN1C1 High/Low expression in (A) TCGA and (B) CGGA and (C) Gravendeel dataset; **(D)** Univariate Cox proportional hazards regression analysis of variables influencing overall survival of GBM patients in the TCGA, CGGA, and Gravendeel dataset.

**Supplementary Figure 4.** MAN1C1, CD133, and CD44 mRNA expression and functional annotation of cluster 11 and 20 in spatial transcriptomics analysis. **(A-C)** Basal mRNA levels of MAN1C1 (A), CD133 (B) and CD44 (C) in proneural GSC11 and mesenchymal GSC20 cells. **(D-E)** Dot plot of the functional enrichment result in cluster 11 (D) and cluster 20 (E) in spatial transcriptomics.

**Supplementary Figure 5.** Functional enrichment and WGCNA analysis in glioma samples. **(A)** Dot plot of the functional enrichment result of MAN1C1 correlated genes in the TCGA_GBM dataset; **(B)** Ridge plot of the GSEA cancer hallmarks correlated with high MAN1C1 in the TCGA_GBM dataset; **(C)** Clustering dendrogram of samples with trait heatmap; **(D)** Analysis of the scalefree index for various soft-threshold powers (β). Left panel, the X-axis represents a function of soft-threshold power, and the Y-axis represents the scale-free fit index. Right panel, the X-axis represents a function of soft-threshold power, and the Y-axis represents the mean connectivity (degree). **(E)** Clustering dendrogram of genes based on the measurement of dissimilarity (1-TOM).

**Supplementary Figure 6.** scRNA-seq analysis in glioma samples. **(A)** DimPlot of different glioma cell clusters. **(B-H)** FeaturePlot of (B) MES2, (C) AC-like (D) OPC-like, (E) NPC1-like, (F) NPC1-like, (G) G1/S, and (H) G2/M enrichment in the glioma cell cluster. **(I-L)** DotPlot of (I) N-glycosylation-related genes, (J) Mucins, Syndecans, Selectins, Selectin ligands, Galectins, Cadherins, MMPs, (K) Integrin subunit genes, and (L) Genes encoding glycosylation targets, components interacting with glycosylation, and components of cancer pathways that regulate or to are affected by glycosylation in different cell clusters.

**Supplementary Figure 7.** Relationship of MAN1C1 expression and immune response. **(A-B)** The TIDE score and response results to immunotherapy of patients with glioma. **(C)** T cell dysfunction, **(D)** T cell exclusion, and **(E)** MDSC, **(F)** CAF, and **(G)** M2 TAM infiltration in high and low MAN1C1 patients. **(H)** Correlation coefficient between MAN1C1 expression and immune checkpoints.
