# Supplementary material for: Elevated α-1,2-mannosidase MAN1C1 in glioma stem cells and its implications for immunological changes and prognosis in glioma patients
Source: Sci Rep. 2024 Sep 27;14:22159. doi: 10.1038/s41598-024-72901-2 (PMC11436702; doi:10.1038/s41598-024-72901-2)

Supplementary Figure 1

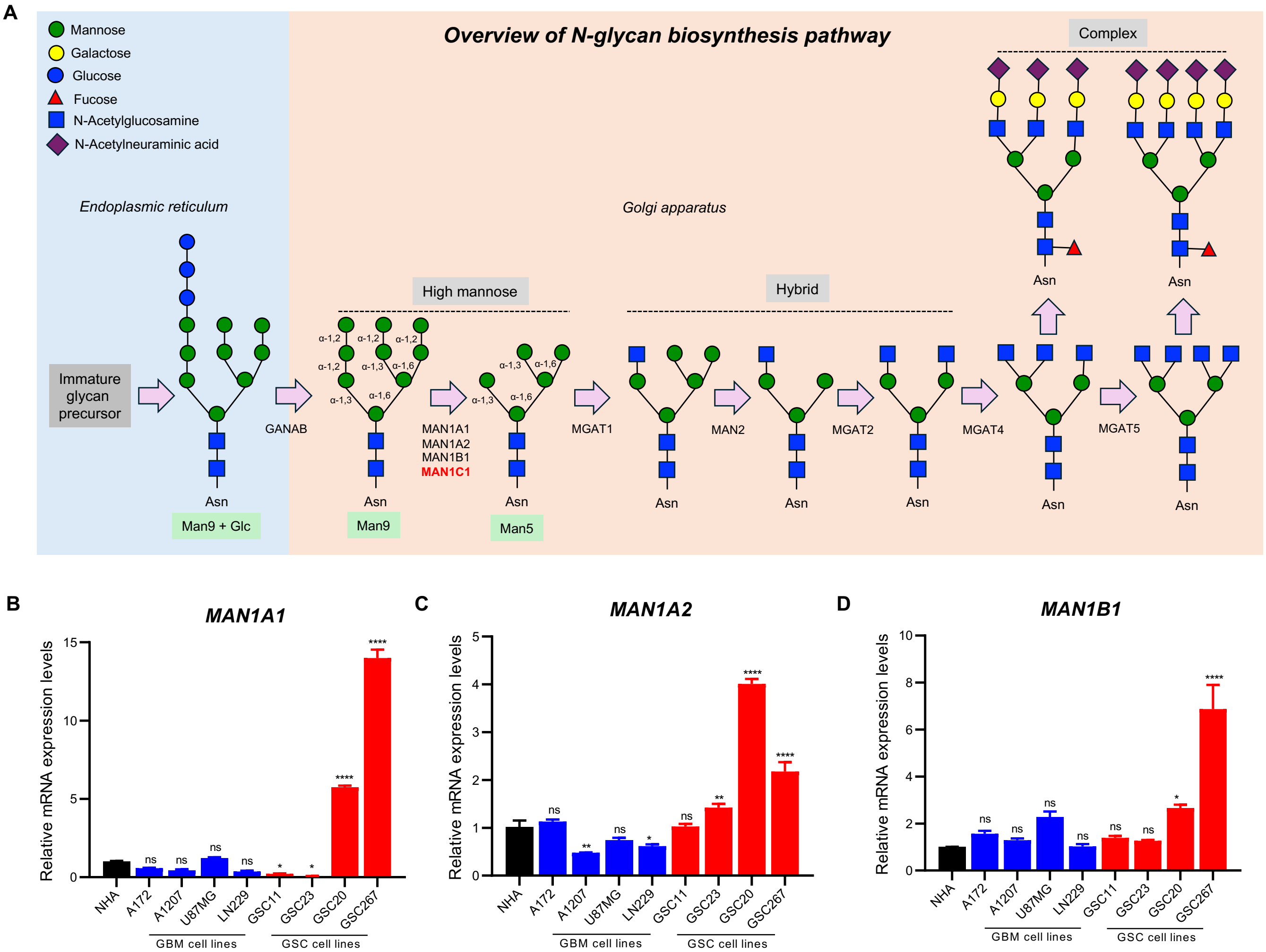

Supplementary Figure 2

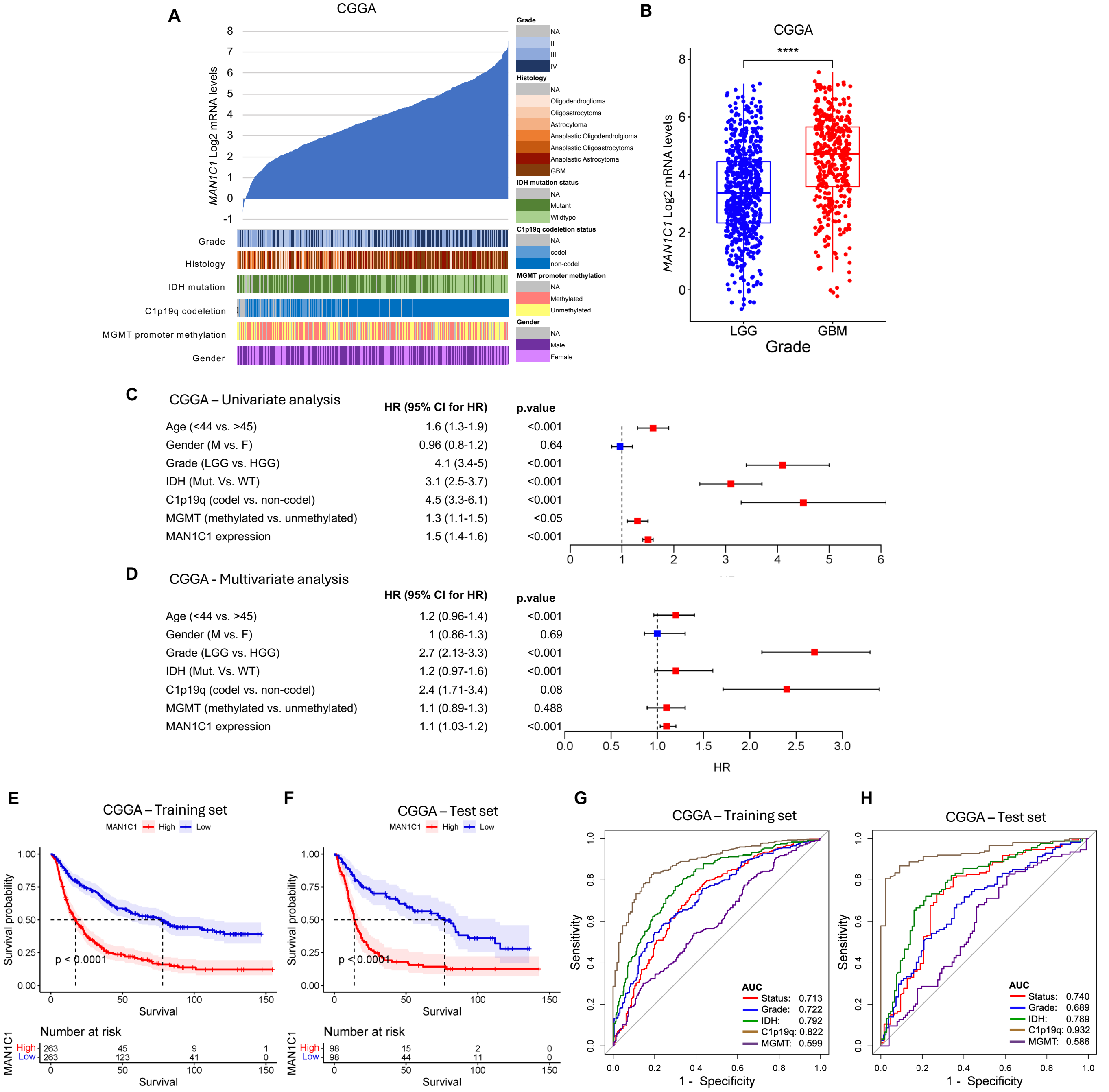

Supplementary Figure 3

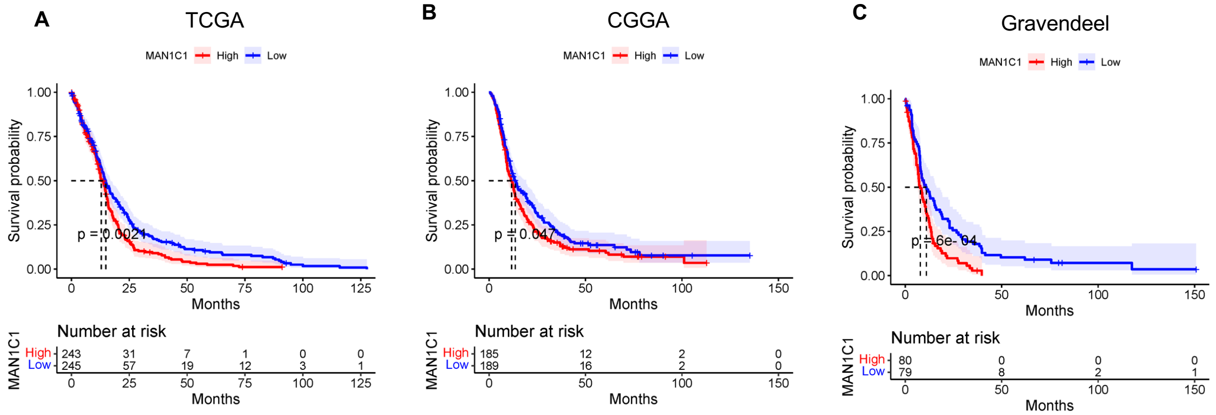

D

	Pvalue	HR
TCGA	0.004	1.100(1.000- 1.200)
CGGA	0.006	1.100(1.000- 1.200)
Gravendeel	0.002	1.300(1.100- 1.500)
Overall	<0.001	1.150(1.100- 1.200)

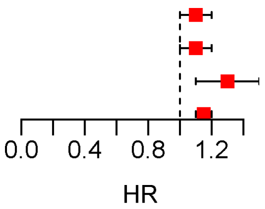

Supplementary Figure 4

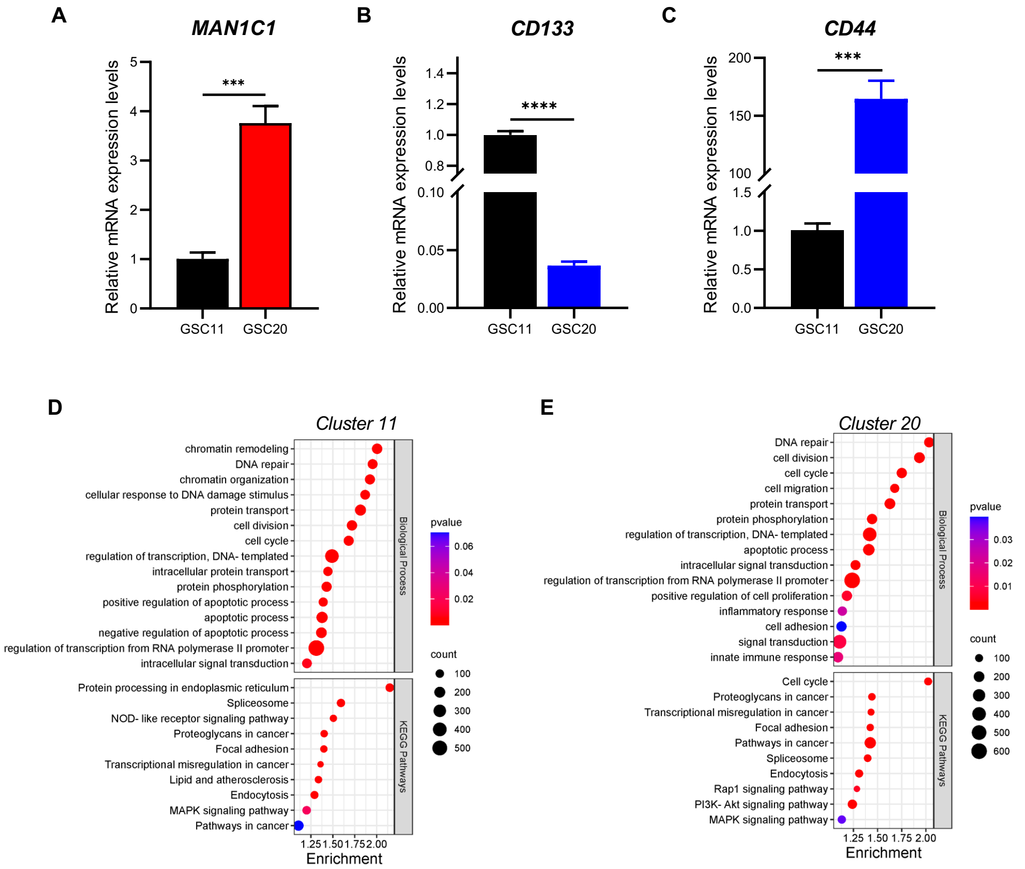

Supplementary Figure 5

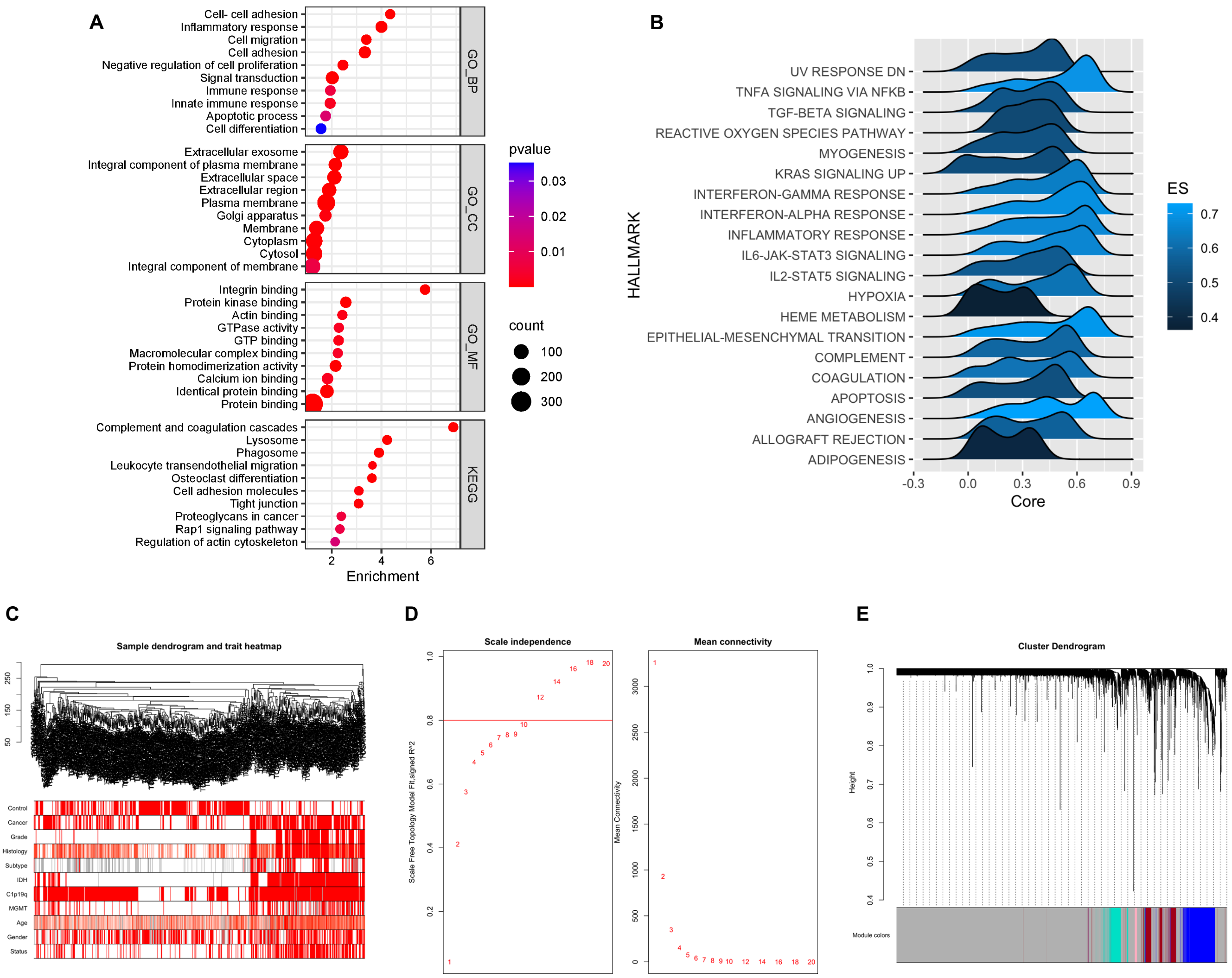

Supplementary Figure 6

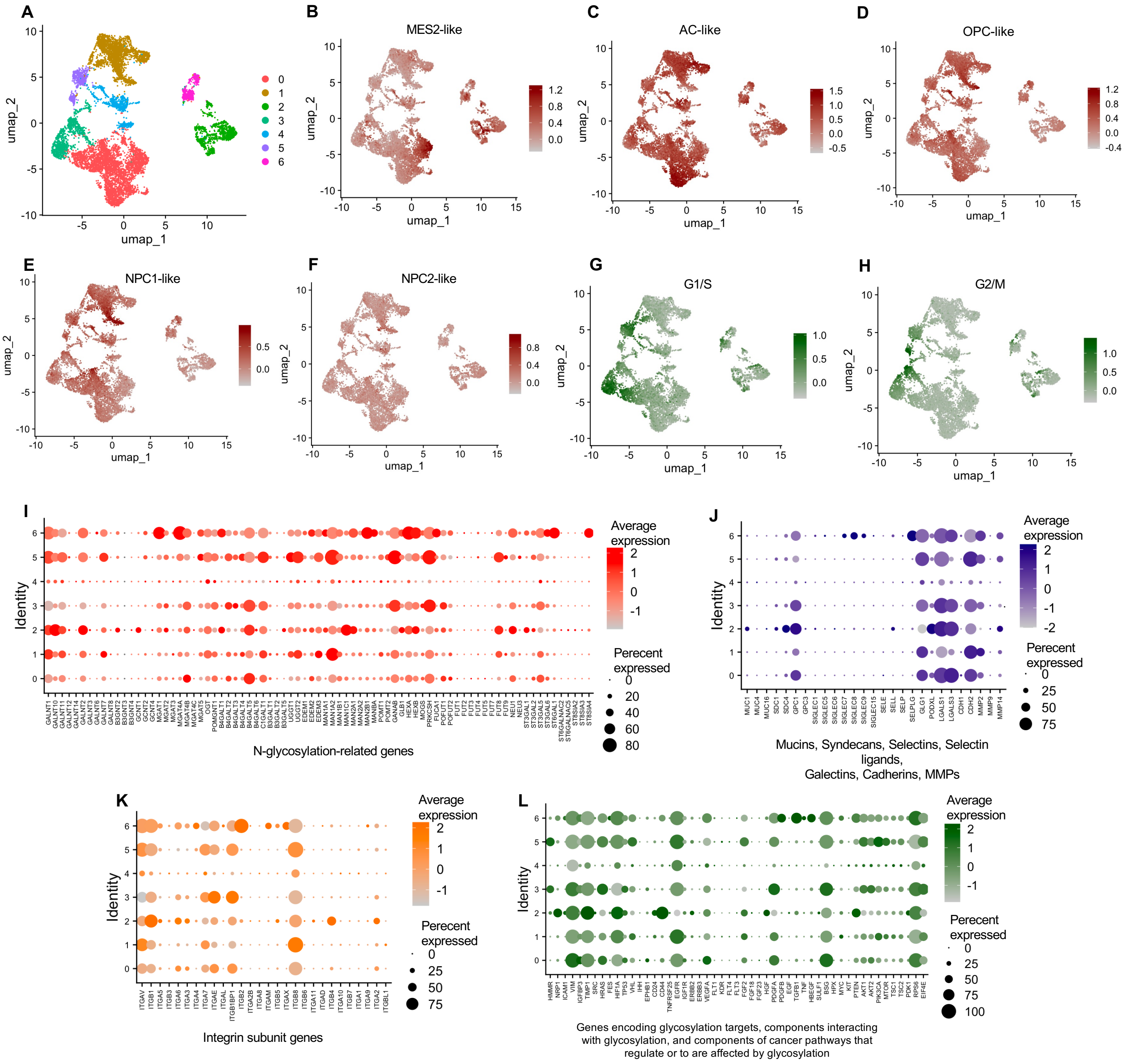

Supplementary Figure 7

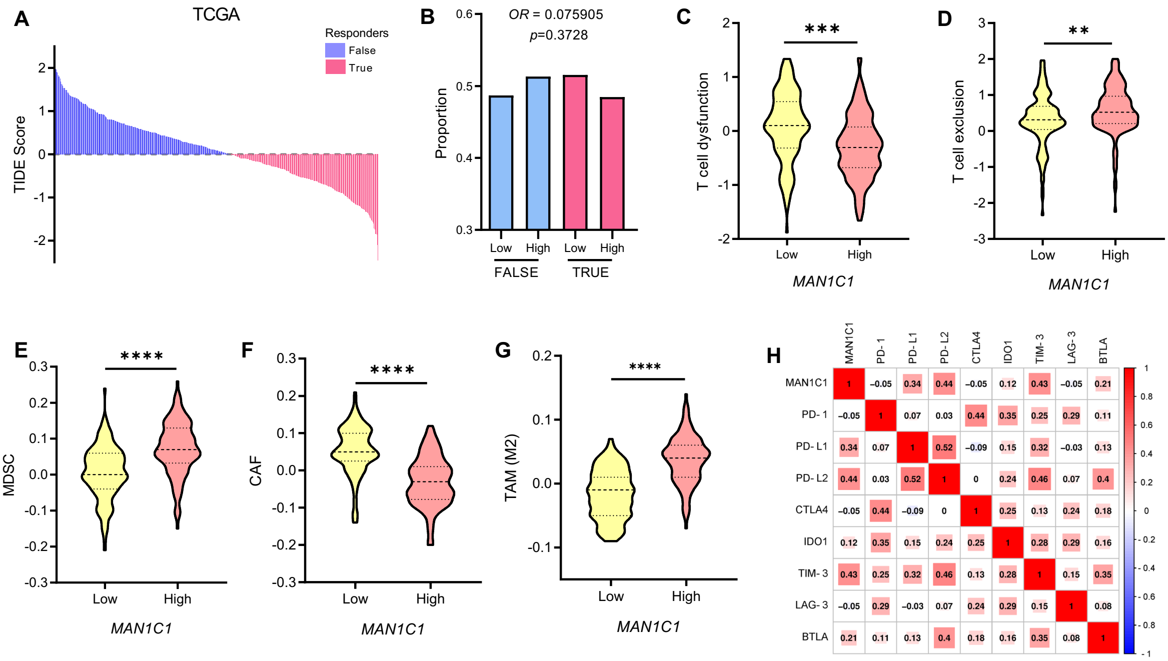

Supplement: Supplementary file 3 — Supplementary Material 3 [file 41598_2024_72901_MOESM3_ESM.pdf]
